# Distinct Microbiomes of Gut and Saliva in Patients With Systemic Lupus Erythematous and Clinical Associations

**DOI:** 10.3389/fimmu.2021.626217

**Published:** 2021-07-01

**Authors:** Fengping Liu, Tianli Ren, Xiaodi Li, Qixiao Zhai, Xifeng Xu, Nan Zhang, Peng Jiang, Yaofang Niu, Longxian Lv, GuoXun Shi, Ninghan Feng

**Affiliations:** ^1^ Wuxi School of Medicine, Jiangnan University, Wuxi, China; ^2^ Department of Urology, Affiliated Wuxi No. 2 Hospital, Nanjing Medical University, Wuxi, China; ^3^ Department of Rheumatology, Affiliated Wuxi No. 2 Hospital, Nanjing Medical University, Wuxi, China; ^4^ State Key Laboratory of Food Science and Technology and School of Food Science and Technology, Jiangnan University, Wuxi, China; ^5^ Department of Outpatient, Wuxi Children’s Hospital of Nanjing Medical University, Wuxi, China; ^6^ Research and Development Department, Hangzhou Guhe Information and Technology Company, Hangzhou, China; ^7^ Collaborative Innovation Center for Diagnosis and Treatment of Infectious Diseases, State Key Laboratory for Diagnosis and Treatment of Infectious Diseases, The First Affiliated Hospital, School of Medicine, Zhejiang University, Hangzhou, China

**Keywords:** complement, disease activity, feces, microbiome, saliva, systemic lupus erythematosus

## Abstract

Alterations in the microbiome of the gut and oral cavity are involved in the etiopathogenesis of systemic lupus erythematosus (SLE). We aimed to assess whether both microbiome compositions in feces and saliva were specific in patients with SLE. A total of 35 patients with SLE, as well as sex- and age-matched asymptomatic subjects as healthy control (HC) group were recruited. Fecal swabs and saliva samples were collected from the participants. 16S ribosomal RNA gene sequencing was performed on the samples. Compared with the HC group, reduced bacterial richness and diversity were detected in the feces of patients with SLE, and increased bacterial diversity in their saliva. Both feces and saliva samples explained the cohort variation. The feces were characterized by enrichment of *Lactobacillus*, and depletion of an unclassified bacterium in the Ruminococcaceae family and *Bifidobacterium*. Lack of *Bifidobacterium* was observed in patients with arthritis. *Akkermansia* and *Ruminococcus* negatively correlated with the serum levels of C3. In saliva, *Veillonella*, *Streptococcus*, and *Prevotella* were dominant, and *Bacteroides* was negatively associated with disease activity. These findings can assist us to comprehensively understand the bacterial profiles of different body niches in SLE patients.

## Introduction

Systemic lupus erythematosus (SLE) is an autoimmune disease in which the immune system attacks its own tissues, causing widespread inflammation and tissue damage in multiple organs. Although the cause of SLE remains unclear, it is thought that hormonal, environmental, and genetic factors are involved. Microbiome, as one of environmental factors, has been suggested to contribute to the occurrence and development of SLE (1, 2).

To date, several studies have described the characteristics of disrupted gut microbiome in patients with SLE. In a cross sectional study, Hevia et al. reported a lower Firmicutes/Bacteroidetes (F/B) ratio in the gut of individuals with SLE, and a decrease in some Firmicutes families (3). Similarly, He et al. also showed depletion of Firmicutes and enrichment of Bacteroidetes in the gut in patients with SLE (1). Azzouz et al. presented clear evidence of gut dysbiosis with a five-fold increase in the abundance of *Ruminococcus gnavus* in patients with SLE versus HCs; this relative abundance were correlated with the SLE disease activity index (SLEDAI) (4). Coincidentally, Li et al. reported that the disrupted microbiome, such as *Streptococcus*, *Campylobacter*, and *Bifidobacterium*, were correlated with the SLEDAI (5). Gut dysbiosis is associated with SLE, and oral dysbiosis contributes to the disease. A recent study reported that subgingival dysbiosis in patients with SLE was characterized by decreased microbial diversity and accompanied by higher proportions of *Fretibacterium*, *Prevotella* and *Selenomonas* (2).

The complement system is an important effector pathway of innate immunity and plays a major role in SLE. It involves a series of proteins that assemble in domino fashion to destroy bacteria invading the body (6). Recent studies demonstrated that the bacterial profile could modulate the complete system in health disorders (7, 8). Hence, alterations in microbiomes may contribute to the production of complements in patients with SLE.

In this study, we recruited patients with SLE, and sex- and age-matched HCs to investigate: (1) whether patients with SLE experience bacterial disturbances in multiple body niches, including gut and oral cavity, at the same time-point; and (2) whether the bacterial disturbances were associated with clinical findings, such as disease activity and the levels of serum complement (C) 3 and C4. The present study may lead to a comprehensive understanding of the contribution of “multi-microbiomes” within the same cohorts of patients and HCs, and aid in microbiome-based diagnosis and treatment.

## Materials and Methods

### Patient Recruitment

The ethics committee of the Affiliated Wuxi Second Hospital of Nanjing Medical University approved this study (Ref. 201805). Informed consent was provided by all subjects prior to sample collection. The sample size of the present study was calculated using data from a previous study on the human urinary microbiome and HMP R-package created by Mattiello et al. (9, 10). Based on sample size which can detect alterations in the gut or saliva microbiome when comparing patients with SLE and HCs in previous studies and a power of 0.90 can be achieved (11–13), 35 patients with SLE, and 35 sex- and age-matched HCs were recruited in the present study.

The inclusion criteria were: aged ≥ 18 years; patients with SLE who fulfilled the American College of Rheumatology classification criteria for SLE (14); patients with active and remissive SLE; and patients currently receiving low-dose prednisone (maximum: 7.5 mg daily) and hydroxychloroquine. Prednisone and hydroxychloroquine are standard treatment options for SLE (11), and previous studies with large sample sizes did not demonstrate a correlation between the use of oral corticosteroid or hydroxychloroquine and changes in gut microbiome (15, 16). The sex- and age-matched healthy subjects who were free of SLE and any other autoimmunity diseases were recruited as HC cohort. The exclusion criteria for either SLE or HC cohort were: presence of diarrhea, constipation, and oral disease or ulceration at inclusion; other autoimmune diseases, pregnancy, breastfeeding, menstruation, recent severe illness or infections, diagnosis of neoplastic disease, use of antibiotics/probiotics/vitamin D and B12/calcium/oral contraceptive/metformin/antibiotics/proton pump inhibitors within the past 2 weeks prior to participation in the study (15–17); current use of immunosuppressive drugs; and non-local Han Chinese residents. Disease activity was scored using the composite SLEDAI (18). As there are various classifications of disease severity in previous studies and clinical settings, we applied two types of classification to assess the influence of disease severity on bacterial community: a) Low disease activity subgroup (LDA; SLEDAI < 6) and High disease activity subgroup (HDA; SLEDAI ≥ 6) (19, 20); b) subgroups of Mild (SLEDAI ≤ 4), Moderate (SLEDAI = 5-8) and Severe (SLEDAI > 8). Renal disorder was defined as: a) Persistent proteinuria greater than 0.5 grams per day or greater than 3+ if quantitation not performed OR b) Cellular casts-may be red cell, hemoglobin, granular, tubular, or mixed (21). Information on clinical manifestations was obtained by reviewing clinical records. In addition, a control cohort of HCs did not have history of autoimmune diseases, diabetes and cancers.

### Sample Collection and DNA Isolation

Fresh fecal material was collected in a sterile container, and 30 mg were placed in a sterile container. The participants were asked to wash their mouth with bottled water to remove food debris, refrain from eating and drinking for 1 h prior to the collection of the saliva sample (13), and at least 1 mL saliva was collected into a sterile tube. Of note, 500 µL of lysis buffer was added to the tube of the saliva sample prior to collection. All samples were immediately stored at −80°C until further processing.

Sera-Mag SpeedBeads Carboxylate-Modified Magnetic Particles (GE Healthcare UK, Little Chalfont, UK) were used to extract the DNA from the feces, and saliva samples, as previously described (22). The quantity and quality of the extracted DNA were measured using a NanoDrop ND-1000 spectrophotometer (Thermo Fisher Scientific, Waltham, MA, USA) and agarose gel electrophoresis, respectively. Polymerase chain reaction (PCR) amplification of the bacterial 16S rRNA genes V3-V4 region was performed using the universal primers 319F and 806R with 30 cycles. PCR amplicons were purified with Agencourt AMPure XP Beads (Beckman Coulter, Indianapolis, IN, USA) and quantified using the PicoGreen dsDNA Assay Kit (Invitrogen, Carlsbad, CA, USA). Following the individual quantification step, amplicons were pooled in equal amounts, and pair-end 2×300 bp sequencing was performed using the Illlumina MiSeq platform at GUHE Info Technology Co., Ltd (Hangzhou, China). Three negative controls consisting of normal saline were used to assess the contribution of contaminating DNA from the reagents, and six negative controls without template DNA were included in the sequencing process.

Blood samples were collected on the day of collection of feces and saliva samples. An immunoturbidimetric test was used to assess the serum levels of C 3 and C4, as well as antibodies in blood (AU5421; Beckman Coulter).

### Bioinformatic Analysis

R1 and R2 paired reads were trimmed at both 3’ and 5’ ends using the Cutadapt v.2018.4.0 software, filtered for base quality (Q>30), and merged (23). FASTQ sequencing data were processed using the open-source bioinformatics pipeline Quantitative Insights Into Microbial Ecology 2 (QIIME 2; v. 2020.2) at the default setting (24). The reads were separately processed with DADA2 to reconstruct the original amplicons (25). The remaining high-quality reads were de-replicated to obtain a unique sequence (uniques) and chimeric sequences were removed using the QIIME DADA2 denoise-paired command. Denoised sequences with ≥ 99% identity versus uniques were *de novo* clustered into Amplicon Sequence Variants (ASV) and summarized in an ASV-by-sample abundance matrix (23). Only the ASVs that represented ≥ 0.005% of the total reads were maintained. Taxonomy was assigned, down to the species level, using Feature Data [Sequence] artefact against the reference database Greengenes V.13-8 (23). Contaminant sequences (based on the negative controls) were removed using Decontam v.1.2.1 with *p* < 0.10 as the threshold.

Alpha diversity was calculated based on a rarefied feature table (rarefied at the lowest sample size) abundance-based coverage estimators (ACE), Chao 1, Shannon, and Simpson’s index. Beta diversity analysis was performed to evaluate differences in species complexity between samples. We applied the permutational multivariate analysis of the variance method to the Bray–Curtis distance data using 999 permutations to analyze feature differences between patients with SLE and HCs; statistical significance was defined as *p* < 0.05 (R software vegan package). Based on the feature abundances, an Upset diagram was used to display the numbers of microbial features shared by the various groups (26).

Fisher’s exact test or Student’s *t* test were applied using SPSS version 24.0 (IBM Corp., Armonk, NY, USA) to compare the clinical variables between the SLE and HC groups. The Wilcoxon rank-sum test was used to compare alpha diversity indices, bacterial abundances, and the F/B ratio between groups. R (version 3.6.2) was used for comparative statistics, and a Benjamini–Hochberg false discovery rate corrected *q*-value was calculated for comparative tests. A *q*-value < 0.05 was used as cut-off for comparative statistical tests. Pearson’s correlation analysis was used to assess the correlations between the relative abundances of bacterial genera and serum levels of C3 and C4 in the samples; correlations with *p* < 0.05 were considered significant.

Sequencing data from this study have been deposited in the GenBank Sequence Read Archive under accession number PRJNA629055 (https://dataview.ncbi.nlm.nih.gov/objects?linked_to_id=SRR11639838&archive=bioproject).

## Results

### Clinical Variables

As shown in **Tables 1**, **S1**–**S3**, samples were collected from 35 patients with SLE, and sex- and age-matched HCs. Their age ranged from 22 to 67 years old. Three of the total participants were male. The disease duration ranged 2 months–20 years, and the SLEDAI ranged 1–12. Thirty patients received hydroxychloroquine and prednisone. As expected, reduced levels of C3 and C4 were detected in the SLE group (p < 0.05). Interestingly, only one SLE patient had decreased GFR, and 3 patients had increased blood uric acid in the present study.

**Table 1:** Demographic, clinical and immunological features of SLE patients.

Patient no.	Age range(yrs)	Disease duration(yrs)	SLEDAI	Complement C3 (g/L)	Complement C4 (g/L)	Clinical manifestations and immunological features	BMI (kg/m^2^)	Hypertension	Type 2 diabetic mellitus
SLE1	40-45	7	6	0.85	0.14	HD, MR	24.77	0	0
SLE2	26-30	0.17	6	0.35	0.05	AR, AMA-M2, anti-P antibodies, anti-SSa, HD, RF	17.71	0	0
SLE3	20-25	0.25	2	0.66	0.17	anti-RNP, anti-RO-52, anti-Sm, anti-SSa, HD, RF	17.69	0	0
SLE4	40-45	10	6	0.96	0.17	AR, HD	19.92	0	0
SLE5	50-55	12	6	0.56	0.08	AHA, ANuA, anti-RNP, anti-RO-52, anti-Sm, anti-SSa, DL, MR	20.00	0	0
SLE6	30-35	3	7	0.80	0.15	/	16.94	0	0
SLE7	20-25	2	6	0.90	0.20	ANA, anti-SSb, HD, MR	29.73	0	0
SLE8	30-35	10	10	0.99	0.16	DL, MR	20.83	0	0
SLE9	46-50	12	5	0.63	0.11	ANA, anti-P antibodies, anti-RNP, anti-Ssa, DL, MR	27.92	0	0
SLE10	56-60	10	5	0.94	0.19	ANA, anti-RNP, anti-SSa, HD	24.80	0	0
SLE11	46-50	14	12	0.77	0.10	ANA, anti-RNP, anti-RO-52, AR, HD, MR	20.76	0	0
SLE12	46-50	7	6	1.15	0.31	HD, MR	27.01	0	0
SLE13	30-35	8	8	0.88	0.13	AR, ANA, anti-P antibodies, PH	21.64	0	0
SLE14	36-40	7	9	0.58	0.06	AR, HD	23.14	0	0
SLE15	26-30	1	7	0.16	0.05	ANA, anti-dsDNA, ANuA, anti-RNP, HD, MR	26.35	0	0
SLE16	56-60	3	6	0.80	0.11	ANA, anti-RNP, DL, MR	22.63	0	0
SLE17	50-55	9	5	1.11	0.23	HD	23.14	0	0
SLE18	36-40	2	11	0.83	0.11	ANA, anti-SSa, anti-RO-52, anti-SSb	17.58	0	0
SLE19	50-55	20	1	1.12	0.17	AHA, anti-RO-52, anti-RNP	20.94	1	0
SLE20	30-35	5	9	0.61	0.07	DL, MR, RD	24.46	0	0
SLE21	30-35	1	7	0.75	0.12	anti-RNP, HD, MR	24.34	0	0
SLE22	20-25	1	1	0.48	0.14	anti-P antibodies, anti-RNP, HD, RF	26.90	0	0
SLE23	60-65	10	6	0.73	0.133	AHA, ANA, AR, anti-dsDNA, anti-RO-52, anti-Ssa	27.68	0	0
SLE24	66-70	20	1	0.80	0.15	/	25.89	0	1
SLE25	36-40	16	10	0.80	0.13	ANA, AR, DL, HD, MR	26.67	0	0
SLE26	40-45	7	1	0.80	0.15	/	22.23	0	0
SLE27	50-55	6	11	1.01	0.20	ANA, anti-RNP, AR, HD, MR	23.37	0	0
SLE28	56-60	10	5	0.80	0.15	HD	19.05	1	0
SLE29	40-45	13	10	1.22	0.26	AHA, ANA, anti-dsDNA, AR, DL	25.00	0	1
SLE30	30-35	9	10	0.80	0.15	HD, MR, SL	16.65	0	0
SLE31	26-30	2	6	0.80	0.15	anti-RO-52, anti-SSb, HD	28.89	0	0
SLE32	46-50	20	9	0.64	0.08	AHA, ANA, ANuA, anti-dsDNA, anti-RO-52, anti-SSa, anti-SSb, DL, HD, MR	23.44	0	0
SLE33	26-30	5	12	1.24	0.31	anti-RO-52, anti-SSb, DL, MR	19.92	0	0
SLE34	50-55	5	5	0.73	0.133	AHA, anti-dsDNA, anti-RO-52, anti-SSa, DL, HD, MR	25.39	0	0
SLE35	36-40	3	3	0.76	0.14	/	22.48	0	0

AHA, anti-histone antibody; ANA, antinuclear antibodies; anti-RNP,Anti-ribonucleoprotein autoantibodies; anti-Sm, anti-Smith antigen antibodies; ANuA,anti-nucleosome antibodies; AR,arthritis; BMI, body mass index; DL, discoid lesions; HD, hematological disorder; MR, malar rash; PH, photosensitivity; RD, renal disorder; RF, rheumatoid factor; SE, serositis; SLEDAI, Systemic Lupus Erythematosus Disease Activity Index.

“0” represents that the participants had not diagnosed with the disease of hypertension or diabetes, whereas “1” represents that the participants were with the disease.

### Microbiome Composition Showed Difference Between Patients With SLE and HCs

The nine negative control samples were sequenced in the present study. 19, 29 and 89 raw reads were detected in the negative control samples without specimens, and 141, 123, 18, 798, 134 and 31 raw reads in the negative control samples without template DNA, respectively. The ASVs yielded in the negative control samples were as follows: 6, 9 and 19 ASVs were detected in the negative control samples without specimens, and 44, 24, 10, 30, 48 and 18 were in the negative control samples without template DNA, respectively. The specific raw reads and ASVs are displayed in **Table S4**.

Of the total 140 samples, 138 samples showed detectable genomic DNA following PCR amplification. Totally, 6,234,764 raw reads were yielded (average raw reads were 45,179; ranged from 27,973 to 64,636); 6,011,261 reads after removing low-quality or ambiguous reads. Goods coverage ranged from 99.41% to 99.99%. The observed features in the group of SLE feces (SLEF), HC feces (HCF), SLE saliva (SLES) and HC saliva (HCS) were 391, 798, 1,208 and 1,107, respectively.

**Figure 1:**
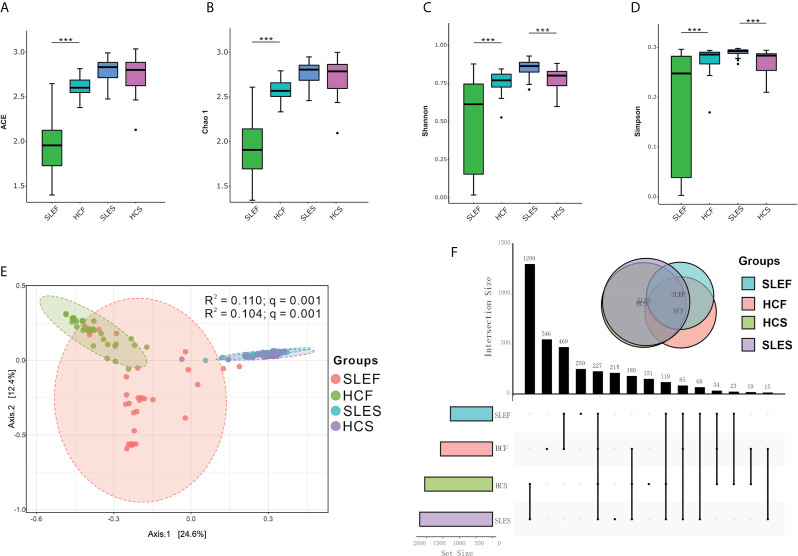
Microbiome compositions in feces and saliva samples obtained from patients with SLE and HC. **(A)** Significantly lower ACE in the feces of the SLE group compared with HC. **(B)** Significantly lower Chao 1 in the feces obtained from the SLE group compared with HC. **(C)** Significantly lower Shannon and higher Shannon in the feces and saliva obtained from the SLE group compared with HC. **(D)** Significantly lower Simpson’s index in the feces, and higher Simpson’s index in the saliva of the SLE group compared with HC. Statistically significant comparisons after the Wilcoxon rank-sum test and Benjamini–Hochberg false discovery rate (FDR) correction between groups are denoted as *0.05; **< 0.01; and ***< 0.001. **(E)** Principal coordinate analysis (PCoA) revealed the clustering of bacterial taxa in the groups based on the Bray–Curtis distance, with each point corresponding to a subject and colored according to the type of sample. Permutational multivariate analysis of variance showed that the separation of bacterial communities in feces and saliva samples was significant (*q* = 0.001), and the disease phenotype explained 11.00% and 10.40% of the variation in the overall bacterial composition of the feces and saliva between the SLE and HC groups, respectively. **(F)** Upset plots illustrating quantitative intersection of the sets of ASVs across the samples. The numbers above the bars show the number of common ASVs between the groups of the samples of SLEF, HCF, SLES and HCS. ACE, abundance-based coverage estimators; HCF, HC feces; HCS, HC saliva; SLEF, systemic lupus erythematosus feces; SLES, systemic lupus erythematosus saliva.

As shown in **Figures 1A–D**, the alpha estimators of richness, such as indices of ACE and Chao 1 were significantly reduced in feces samples in the SLE group versus the HC group (*q* < 0.001). The SLES group tended to have higher bacterial richness indices compared with the HC group; however, the difference was not statistically significant (*q* > 0.05). Similar to the bacterial richness, the bacterial diversity estimators, including the Shannon and Simpson’s indices, were significantly lower in the SLEF group versus the HCF group (*q* < 0.001). The SLES group demonstrated significantly higher values for the Shannon and Simpson’s indices compared with the HC group (*q* < 0.001).

When the SLE patients were divided into subgroups of Low disease activity feces (LDAF; SLEDAI < 6) and High disease activity feces (HDAF; SLEDAI ≥ 6), and subgroups of mild feces (MildF; SLEDAI ≤ 4), moderate feces (ModerateF; SLEDAI = 5-8) and severe feces (SevereF; SLEDAI > 8), there were no differences in bacterial richness and diversity between subgroups of LDAF and LDAF, and among subgroups of MildF, ModerateF and SevereF. In contrast, significantly reduced levels of bacterial richness and diversity in feces were observed in all stages of SLE patients comparing to controls, regardless of the types of classification applied (**Figures S1A, B** **).**

Also, we divided the saliva samples using the abovementioned SLEDAI cut-offs to assess the microbial richness and diversity alterations. The comparison of low disease activity saliva (LDAS; SLEDAI < 6) and High disease activity Saliva (HDAS; SLEDAI ≥ 6) did not show significant difference in bacterial richness. However, both subgroups of LDAS and HDAS, displayed significantly higher levels of bacterial diversity estimators (Shannon and Simpson indices) than those in HCS (**Figure S2A**). Interestingly, the group of mild saliva (MildS) had sharply decreased levels of bacterial richness and diversity than those in moderate saliva (ModerateS) group, while the Milds did not show difference in these estimators comparing with HCS. In addition, the subgroups of ModerateS and severe saliva (SevereS) had increased levels of bacterial diversity than those in HCS group (**Figure S2B**).

The microbial community structures in all samples from the SLE and HC groups could be separated by unweighted UnFrac-based principal coordinates analysis (PCoA), such as between the groups of SLEF and HCF, as well as SLES and HCS (R^2^ =0.110, and 0.104, respectively; both *q* = 0.001; **Figure 1E**). However, when the SLE fecal samples were divided into subgroups of LDAF and HDAF, and subgroups of MildF (SLEDAI ≤ 4), ModerateF (SLEDAI = 5-8) and SevereF (SLEDAI > 8), no significant differences in bacterial community were observed (**Figures S3A, B**). Similar findings were found in the saliva samples (**Figures S4A, B**).

An Upset diagram showed that there were 1,265 features in SLEF and HCF samples, of which 469 (37.08%) were shared by the two groups; there were 1,661 features in the SLES and HCS samples, of which 1,299 (78.21%) were shared by the two groups (**Figure 1F**).

### Bacterial Taxonomy in Feces Samples

At the phylum level, the SLEF and HCF groups were dominated by Firmicutes, Bacteroidetes, Actinobacteria, Proteobacteria, and Fusobacteria (**Figures S5A, B**); however, differences in their abundances were not statistically significant. Moreover, when the F/B ratio was calculated, there was no significant difference between the SLEF and HCF group, between HDAF/LDAF and HCF group (*q* > 0.05; **Figures S6A, B**).

**Figure 2:**
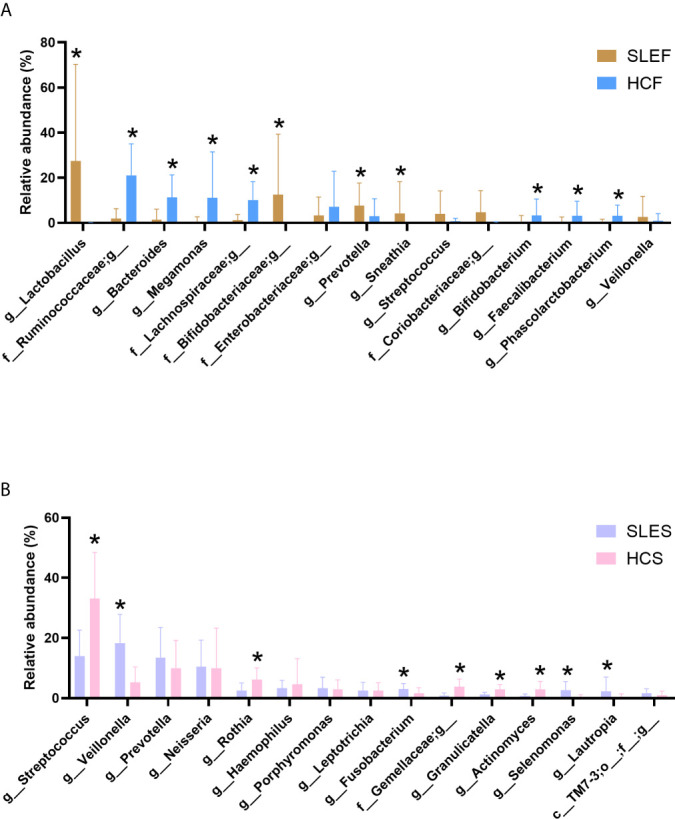
Comparison of the microbiome by cohort. **(A)** The mean sequence abundance of the 15 most abundant bacterial genera in feces was compared. **(B)** The mean sequence abundance of the 15 most abundant bacterial genera in saliva was compared. The class or families Bifidobacteriaceae, Coriobacteriaceae, Enterobacteriaceae, Gemellaceae, Lachnospiraceae, Ruminococcaceae, and TM7-3 could not be classified to the genus level. A Wilcoxon rank-sum test was used to compare the mean sequence abundances between the cohorts. “*” represents *q* values < 0.05. HCF, HC feces; HCS, HC saliva; SLEF, systemic lupus erythematosus feces; SLES, systemic lupus erythematosus saliva.

**Figure 2A** demonstrated that the samples of SLEF were dominated by *Lactobacillus*, an unclassified bacterium in the Bifidobacteriaceae family, *Prevotella*, *Sneathia*, an unclassified bacterium in the Coriobacteriaceae family, etc. In contrast, the HCF group was dominated by a bacterial genus in the Ruminococcaceae family, *Bacteroides*, *Megamonas*, an unclassified bacterium in the Lachnospiraceae family, an unclassified bacterium in the Enterobacteriaceae family, etc.

By comparing the bacterial genus (**Figure 2A**), we observed that the abundance of *Lactobacillus* was sharply increased in the SLEF group than the HCF group (*q* < 0.001). Furthermore, it accounted for > 95% of the total abundance in the following eight samples: SLE2, SLE3, SLE9, SLE15, SLE17, SLE18, SLE25, and SLE29 (**Figure S7**). However, these eight samples did not exhibit special clinical manifestations (**Table 1**). *Lactobacillus* accounted only for 0.02% of the total abundance in the HCF group, and it was not detected in 55.88% (19/34) of samples. Notably, it can be classified as a biomarker for patients with SLE in fecal samples (**Figures S8A, B**). In addition, *L. iners* exhibited significantly higher levels in the SLEF group versus the HCF group (41.48 ± 47.68 vs. 0.00 ± 0.01, respectively; *q* = 0.007). A significantly declined Ruminococcaceae family was observed in the SLEF group compared with the HCF group (2.36 ± 5.71 vs. 26.93 ± 18.43, respectively; *q* < 0.001). Interestingly, an unclassified bacterium which accounted for 99.56% of the total abundance of the Ruminococcaceae family was significantly reduced in the SLEF group (**Figure 2A**). Moreover, it was not detected nearly half of the samples in the SLEF group, including SLE2, SLE4, SLE5, SLE13, SLE18–SLE21, SLE24–SLE29, and SLE32 (**Figure S7**). However, there were no associations with clinical manifestations in these patients. *Bifidobacterium* was sharply decreased in the SLEF group versus the HCF group (*q* < 0.05), and was not detected in 58.82% (20/34) of samples, including SLE2–SLE5, SLE8, SLE13–18, SLE20, SLE22, SLE23–SLE25, SLE27–SLE29 and SLE32. Interestingly, we observed that eight of the nine SLE patients with arthritis had no detectable levels of *Bifidobacterium* in the present study (**Table 1** and **Figure S7**). In addition, a significant decrease in *B. adolescentis* and *B. longum* was shown in the SLEF group compared with the HCF group (0.34 ± 1.46 *vs.* 4.33 ± 8.67, respectively; *q* = 0.029 and 0.38 ± 1.58 *vs.* 5.75 ± 8.84, respectively; *q* = 0.010). *Prevotella* was significantly enriched in the SLEF group (*q* < 0.05; **Figure 2A**), and it was a biomarker for distinguishing SLEF samples from HCF samples (**Figures S8A, B**). Additionally, SLEF had increased *Blautia* genus than that in the HCF (0.51 ± 1.29 vs 0.10 ± 0.70; *q* < 0.001).

To assess the influence of disease severity on the levels of bacterial genus in feces, the genus with relative abundance above 1% of the total abundance was compared among subgroups with the abovementioned two types of SLEDAI cut-offs. As **Figure S9** shown, most of the bacterial genera showed statistical difference between LDAF/HDAF and HCF, while *Prevotella* and *Sneathia* showed significant difference between groups of LDAF and HDAF. **Figure S10** displayed that there were no differences among the subgroups of MildF, ModerateF and SevereF at the genus level in feces. And it is worth to note that several bacterial genera only exhibited significant differences between groups of ModerateF/SeverF and HCF, including *Bifidobacterium*, *Lactobacillus*, and *Ruminococcus*, etc.

**Figure 3:**
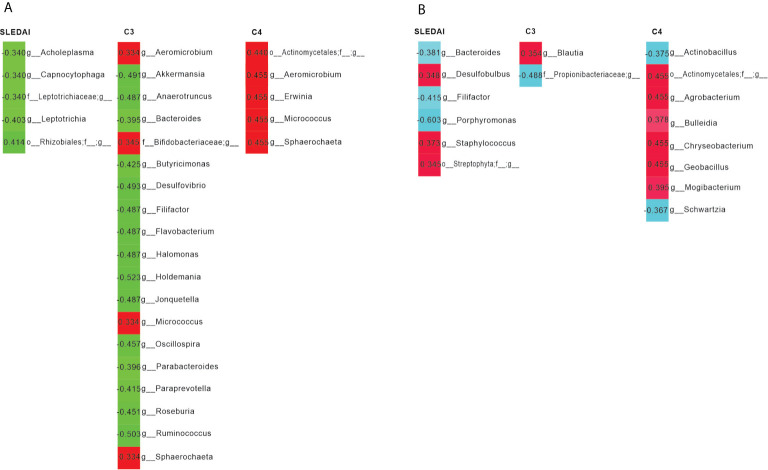
Pearson’s correlation analysis of the bacterial genera and systemic lupus erythematosus disease activity index (SLEDAI), as well as the levels of C3 and C4. **(A)** Bacterial genera in feces were most closely correlated with the SLEDAI, C3 and C4. Positive and negative values of r indicate positive (red) and negative (green) correlations, respectively, between the relative abundance of a genus and the SLEDAI, as well as C3 or C4. Only significant correlations (*p* < 0.05) are shown. **(B)** Bacterial genera in saliva were most closely correlated with the SLEDAI, C3, and C4. Positive and negative values of r indicate positive (red) and negative (light blue) correlations, respectively, between the relative abundance of a genus and the SLEDAI, as well as C3 or C4. Only significant correlations (*p* < 0.05) are shown.

We observed that the SLEDAI score in the SLE group was negatively correlated to the abundance of Acholeplasma, Capnocytophaga and Leptotrichia, etc., and the serum levels of C3 in patients were negatively correlated with the abundance of *Akkermansia*, *Bacteroides*, and *Ruminococcus* in fecal samples (**Figure 3A**).

### Bacterial Taxonomy in Saliva Samples

Among the five most abundant bacteria in saliva, only Actinobacteria were significantly declined in the SLES group compared with the HCS group (*q* < 0.001; **Figures S5C, D** **)**. Similar to feces samples, the saliva samples of patients had reduced abundance of Firmicutes compared with that of healthy subjects (**Figures S5C, D**); however, the difference was not statistically significant (*q* > 0.05). Interestingly, Bacteroidetes and Proteobacteria were slightly elevated in the SLES group versus the HCS group, which was dissimilar to that noted in the feces samples (*q* > 0.05; **Figures S5C, D**).

As shown in **Figure 2B**, the SLES group was composed of *Veillonella*, *Streptococcus*, *Prevotella*, *Neisseria*, *Haemophilus*, etc. In addition, the abundance of *Veillonella* accounted for 20% in nearly half of the samples obtained from patients with SLE (**Figure S11**); however, this high abundance was not associated with clinical manifestations. Among the predominant bacteria in the SLES group, we observed a statistically significant decrease in *Streptococcus* in the SLES group compared with the HCS group (*q* < 0.001; **Figure 2B**), and *S. anginosus* was also significantly declined in the SLES group versus the HCS group (0.98 ± 1.21 vs. 5.71 ± 9.70; *q* = 0.010). In addition, both the Wilcoxon rank-sum test and linear discriminant analysis effect size analysis showed that *Prevotella*, *Selenomonas*, and *Veillonella* were significantly enriched in the SLES group (**Figures S12A, B**).

Similar to fecal samples, the bacterial genus with relative abundance was above 1% of the total abundance was compared based on SLEDIA score of the patients. As **Figure S13** shown, the bacterial genera exhibited non-significant difference between LDAS and HDAS, and among MildS, ModerateS and SevereS (**Figure S14**). Notably, all of the bacterial genera did not show significant difference between MildS and HCS group, while all of them showed difference between ModerateS/SevereS and HCS group except for *Lautropia* (**Figure S14**).

Interestingly, we found that *Bacteroides* was negatively linked to the SLEDAI of patients, while *Staphylococcus* was positively associated with the SLEDAI. In addition, serum levels of C3 was positively correlated to the abundance of *Blautia*, and C4 was positively associated to *Geobacillus* (**Figure 3B**).

### Bacterial Genus Presence in Samples of Feces and Saliva

To demonstrate the bacterial propensity in fecal and saliva samples, we listed the classified bacterial genus in **Tables S5** **,** **S6**. As **Table S5** shown, the distinct bacterial genera in SLEF and SLES were 28 and 22, respectively. And the shared bacterial genera in SLEF and SLES were 104. In the meanwhile, we found that the distinct bacterial genera in HCF and HCS were 27 and 57, respectively. The fecal and saliva samples in HC subjects shared 83 genera. Notably, several bacterial genera only detected SLEF samples, such as *Ureaplasma*, *Paraprevotella* and *Sphingomonas*, etc, but in HCF samples (**Tables S5** **,** **S6**).

## Discussion

Disrupted gut and oral microbiome composition have been suggested as possible environmental factors in the etiology of SLE (2, 3, 5, 11). The present study showed that both feces and saliva samples had disrupted microbiome composition. Since most of the authors of previous studies were focused on fecal microbiome alterations in SLE patients, here we briefly summarized the main findings of theirs and our present study (**Table S7**).

In terms of the bacterial composition in patients’ gut, the analysis revealed reduced levels of ACE, Chao 1, Shannon index, and Simpson’s index, similar to previous studies (1, 3, 5, 11, 12). Loss of microbial diversity in the gut is common in the unhealthy state (27). Further multicenter studies with large population size are warranted to investigate the cause of bacterial lose in patients with SLE. When the bacterial richness of the saliva was assessed, patients with SLE presented slightly higher levels than HCs. This is similar to the study conducted by Corrêa et al., in which they compared the subgingival microbiome between patients with SLE who were free of chronic periodontitis at inclusion and HCs (2). The similar findings of these studies may be attributed to the absence of oral disease at inclusion (2). However, the saliva microbiome of patients with SLE exhibited significantly higher bacterial diversity in the present study. This finding is consistent with that of a previous study on the Chinese population who were not undergoing anti-SLE treatment (28). These results suggest that anti-SLE treatment, including the administration of hydroxychloroquine or/and prednisone, may not affect the bacterial diversity in the oral cavity.

The value of squares in the PCoA analysis represents that the disease phenotype explained the variation in overall bacterial compositions between the SLE and HC groups, in which declined in the feces samples comparing to the saliva samples. However, an opposite change in the shared observed features was observed in the Upset diagram, with an increase in the saliva samples comparing to the feces samples. These findings suggest that the alteration of microbial composition in the feces is not consistent to the saliva in the same cohort of SLE patients.

Notably, the bacterial compositions in terms of bacterial richness and diversity, as well as the PCoA analysis in feces samples were not significantly different when we divided the patients into subgroups of LDAF and HDAF, and MildF, ModerateF and SevereF; hence, the fecal bacterial profiles were not linked to the severity of the disease in the present study. These findings in the gut demonstrated both similarity and dissimilarity to those of a previous study which compared the difference in the fecal microbiome between patients with active and remissive SLE (5). The similarity is that they also did not show difference in alpha diversity between active and remissive patients. The dissimilarity is that they showed that the active patients were distinctly different from remissive patients in the PCoA analysis (5). Whereas, it seems that the bacterial richness and diversity in saliva were associated to disease activity, since the group of MildS had decreased levels of bacterial richness and diversity than those in controls.

In the study conducted by Hevia et al., reduction of the F/B ratio in fecal samples is considered a characteristic of patients with SLE (3). However, Li et al. reported that the ratio of F/B in Chinese patients with SLE, including patients in the active or remissive status, was not significantly different compared with that calculated in HCs (5). Similar findings were demonstrated in our present study. The difference between subjects in the study conducted by Hevia et al. and our Chinese population suggests that race and location play a partial role in the microbial profile of patients with SLE.

Interestingly, several bacteria presented different alterations in the feces and saliva samples between patients with SLE and HCs. For instance, *Streptococcus* was significantly reduced in patients’ saliva and did not show significant alteration in their feces. Further study is warranted to investigate the difference only presented in the saliva, instead of the feces, which are currently the most investigated body niches in the human microbiome.

Surprisingly, the patients with high abundance of *Lactobacillus* in our present study did not show different clinical manifestations versus those with low levels of this bacterium. However, disease severity might play a role in the high levels of *Lactobacillus* in SLE group, since only patients in the status of moderate and severe had significantly increased abundance of *Lactobacillus* than that in controls. Mu et al. applied a mixture of *L. oris*, *L. rhamnosus*, *L. reuteri*, *L. johnsonii*, and *L. gasser* strains to treat gut dysbiosis in SLE nephritis mice. They found that the mixture of *Lactobacillus* spp. improved the symptoms of lupus. However, *L. reuteri* accounted for most of the observed effects among the strains in the mixture (12). These findings suggest that different strains in *Lactobacillus* may activate different components of the immune response in patients with SLE. In the present study, *L. iners* was elevated in the feces of patients. *L. iners* is an unusual bacterium within the *Lactobacillus* genus, since it contributes to the onset and maintenance of vaginal dysbiosis (4). Therefore, whether *L. iners* contributes to gut disruption in SLE should be investigated.

In the gut, Bacteroides was significantly lower in the SLE group compared with the HC group. The depletion of Bacteroides in the gut was inconsistent with the results of previous studies on patients from Spain and the Netherlands (3, 11).

*Blautia* was sharply increased in SLE patients in our present study. Similar finding was reported by several previous studies on American population using 16S rRNA sequencing and one study in Chinese population using shotgun metagenomics sequencing (11, 29, 30). However, similar finding had not been confirmed by studies on participants from Spain and the Netherlands (1, 3, 31), and studies on Chinese population using 16S rRNA sequencing (5, 32, 33). Thus, multicenter study on populations in various locations and races using the same sequencing methods is required to confirm the correlations between the function of Blautia in SLE.

Although an increase in *Prevotella* was observed in both gut and saliva samples, a significant difference was only detected in fecal samples. Similar research on the gut microbiome compositions of Chinese patients with SLE yielded consistent results with the findings of this study (1).

Notably, the depletion of some bacteria in the gut of patients with SLE because of their disappearance in most of the samples. For example, the reduction in Ruminococcaceae was accompanied by the disappearance of an undetectable unclassified bacterium in more than half of the patients. The reduction in Ruminococcaceae was also shown by a previous study on human SLE (33). However, an opposite change presented in a murine lupus model (34). This inconsistency represents that there may be some difference between observations in human and animal models. Another bacterium, *Bifidobacterium*, had also disappeared in more than half of the samples. In the study conducted by Li et al., *Bifidobacterium* was negatively associated with disease activity (5); however, this association was not observed in the present study. Interestingly, we found that the lack of *Bifidobacterium* is responsible for the occurrence of arthritis in patients with SLE, a finding which has not been reported in previous gut microbiome studies. Accompanied by the reduction of *Bifidobacterium* in the feces of patients with SLE, the levels of *B. adolescentis* and *B. longum* were sharply declined in the patients. *B. longum* has been used as a probiotic supplementation in patients with autoimmune disease (35). Further research should focus on clarifying how *B. longum* produce and the extent of its specific probiotic efficacy in SLE.

Similar to the study performed by Corrêa et al. on the oral microbiome (2), elevation of *Selenomonas*, and depletion of *Haemophilus* and *Streptococcus* in the saliva of patients with SLE was observed in the present study. It appears that the abundance of pathogens was increased in the SLE group. For example, *Selenomonas* is responsible for periodontitis (36). Also, *Veillonella* and *Lautropia* were increasing in the saliva of patients with SLE, which were shown to be more abundant in the plaque of patients with gingivitis in a previous study (37). Notably, it seems that that both *Selenomonas* and *Veillonella* are affected by disease activity. As the present study demonstrated that only moderate and severe patients had higher levels of them comparing to the controls. Furthermore, *Fusobacterium*, which is treated as a pathogen in pharyngitis (38), was also increased in the patient group.

When the correlations between bacterial genus and disease activity, and the levels of serum complements were observed, fecal *Akkermansia* and *Ruminococcus*, usually considered probiotics, were negatively correlated with the serum levels of C3. It is evident that *Akkermansia* spp. are involved in host immunological homeostasis at the gut mucosa and improvement of gut barrier function (39). Furthermore, *Ruminococcus* spp. can restore the number of T regulatory cells, which provides a rationale for the use of probiotic therapy (40). *In vivo* experiments are needed to re-assess this negative connection and underlying mechanisms. In the saliva, disease activity was negatively correlated with *Bacteroides*, which is responsible for individuals with active caries (41). These findings suggest that this bacterium plays a pathogenic role in human oral health and its abundance can be used as an indicator for the assessment of disease severity.

Comparing the findings in previous studies and our present study, it seems that ethnicities and participants’ locations play a partial role in SLE patients’ gut microbiome profile. For instance, the declined F/B ratio was reported by two studies on Spanish patients, one study on Netherlands patients, as well as one study on Chinese patients, whereas this finding had not confirmed by studies on American patients and the remaining studies on Chinese patients (1, 3, 5, 29–33). In addition, the decreased bacterial richness and diversity was no described by all previous studies. In Hevia A et al. study, there was a comparable Shannon index in the groups of patients and controls (3). It is recommended that a multicenter study in which patients and controls from various ethnicities and locations are recruited.

## Conclusions

Overall, the present study described that microbial disruption can be observed at body niches in patients with SLE, such as feces and saliva. The most common microbes that comprise the gut and saliva microbiome tended to be distinct from each other. However, they are all associated with SLE and some of the members of the genera in patients responded to clinical characteristics, such disease duration, disease active, and serum levels of C3 and C4. These findings, based on the comprehensive analysis of microbiome profiles in multiple body niches, may aid in the diagnosis and treatment of this disease.

Our study has two limitations to consider. First, the present study was based on Chinese populations, and the sample size of the validation cohort was relatively small. Second, patients currently receiving prednisone, hydroxychloroquine were not excluded, since they are standard treatment of SLE. Future study should include new onset patients who have never been administered medication to rule out the impact of medication on human microbiome.

## Data Availability Statement

The datasets presented in this study can be found in online repositories. GenBank Sequence Read Archive underaccession number PRJNA629055.

## Ethics Statement

The ethics committee of the Affiliated Wuxi Second Hospital of Nanjing Medical University approved this study (Ref. 201805). Informed consent was provided by all subjects prior to sample collection. The patients/participants provided their written informed consent to participate in this study.

## Author Contributions

Conceptualization: NF, GS, LL, and FL. Methodology: TR, XL, QZ, NZ, PJ, and YN. Software: YN, FL, and QZ. Validation: FLand NF. Formal analysis: FL. Investigation: NZ and PJ. Resources: NF and XX. Data curation: FL, NZ, TR, and GS. Writing-original draft preparation: FL. Writing-review and editing: FLand QZ. Visualization: FL. Supervision: FL, LL, and NF. Project administration: NF and FL. Funding acquisition: NF. All authors contributed to the article and approved the submitted version.

## Funding

This research was funded by the Municipal Social Development Science and Technology Demonstration Project in 2019 (grant number: N20192002).

## Conflict of Interest

Author YN was employed by the company Hangzhou Guhe Information and Technology Company.

The remaining authors declare that the research was conducted in the absence of any commercial or financial relationships that could be construed as a potential conflict of interest.

## Supplementary Material

Supplementary Figure 1 Bacterial richness and diversity in feces samples of subgroups. **(A)** Bacterial richness and diversity index compared in fecal samples among LDAF, HDAF and HCF; **(B)** Bacterial richness and diversity index compared in fecal samples among MildF, ModerateF and SevereF. Statistically significant comparisons after the Wilcoxon rank-sum test and Benjamini–Hochberg false discovery rate (FDR) correction between groups are denoted as *0.05; ** < 0.01; and *** < 0.001. HCF, HC feces; HDAF, High Disease activity feces; LDAF, Low disease activity feces; MildF, mild feces; ModerateF, moderate feces; SevereF, severe feces.

Supplementary Figure 2 Bacterial richness and diversity in saliva samples of subgroups. **(A)** Bacterial richness and diversity index compared in saliva samples among LDAS, HDAS and HCS; **(B)** Bacterial richness and diversity index compared in saliva samples among MildS, ModerateS and SevereS. Statistically significant comparisons after the Wilcoxon rank-sum test and Benjamini–Hochberg false discovery rate (FDR) correction between groups are denoted as * 0.05; ** < 0.01; and *** < 0.001. HCS, HC saliva; HDAS, High disease activity saliva; LDAS, Low disease activity saliva; MildS, mild saliva; ModerateS, moderate saliva; SevereS, severe saliva.

Supplementary Figure 3 Principal coordinate analysis (PCoA) in fecal samples of subgroups. PCOA analysis revealed the clustering of bacterial taxa in the groups based on the Bray–Curtis distance, with each point corresponding to a subject and colored according to the type of sample. HCF, HC feces; HDAF, High Disease activity feces; LDAF, Low disease activity feces; MildF, mild feces; ModerateF, moderate feces; SevereF, severe feces.

Supplementary Figure 4 Principal coordinate analysis (PCoA) in saliva samples of subgroups. PCOA analysis revealed the clustering of bacterial taxa in the groups based on the Bray–Curtis distance, with each point corresponding to a subject and colored according to the type of sample. HCS, HC saliva; HDAS, High disease activity saliva; LDAS, Low disease activity saliva; MildS, mild saliva; ModerateS, moderate saliva; SevereS, severe saliva.

Supplementary Figure 5 Microbiome composition in the feces samples of SLE patients and HCs **(A, B)**, and saliva samples of SLE patients and HCs (C and D) at the phylum level. Only the 5 most abundant bacterial phylum are displayed. HCF, HC feces; HCS, HC saliva; SLEF, systemic lupus erythematosus feces; SLES, systemic lupus erythematosus saliva.

Supplementary Figure 6 Box plot of the comparison of Firmicutes/Bacteroidetes ratios between groups (median ± IQR). **(A)** Ratio between the SLEF and HCF groups. **(B)** Ratio among HDAF, LDAF and the HCF group. F/B ratio, Firmicutes/Bacteroidetes ratio; HDAF, High Disease activity feces; IQR, interquartile range; LDAF, Low disease activity feces; SLE, systemic lupus erythematosus.

Supplementary Figure 7 Fecal microbiome profile by cohort based on 16S rRNA gene V3-V4 sequencing. Stacked bar plots depict the sequence abundances of the 15 most abundant genus level taxa in the SLEF and HCF individuals. The remainder of sequences were combined in the category labeled Other. Taxa were ranked according to the mean abundance across all samples. HCF, healthy control feces; SLEF, systemic lupus erythematosus feces.

Supplementary Figure 8 SLEF-specific biomarkers. Linear discriminant analysis Effect Size (LEfSe) shows differentially abundant features as biomarkers determined using the Kruskal–Wallis test (*p* < 0.05) with a LDA score > 3.5. Observed features with different abundance in the feces of patients comparing to the HC. **(A)** LEfSe cladogram demonstrating microbiome differences at various phylogenic levels; **(B)** LEfSe analysis with linear discriminant analysis (LDA) score representing statistical and biological differences at various phylogenic levels between groups. HCF, HC feces; SLEF, systemic lupus erythematosus feces.

Supplementary Figure 9 Comparison of bacterial genus in feces using SLEDAI score 6 as a cut-off. Statistically significant comparisons after the Wilcoxon rank-sum test and Benjamini–Hochberg false discovery rate (FDR) correction between groups are denoted as *0.05; ** < 0.01; and *** < 0.001. HDAF, high disease activity feces; HCF, HC feces; LDAF, low disease activity feces.

Supplementary Figure 10 Comparison of bacterial genus in feces when dividing SLE patients into mild, moderate and severe group. Statistically significant comparisons after the Wilcoxon rank-sum test and Benjamini–Hochberg false discovery rate (FDR) correction between groups are denoted as *0.05; **< 0.01; and ***< 0.001.. HCF, HC feces; MildF, mild feces; ModerateF, moderate feces; SevereF, severe feces.

Supplementary Figure 11 Salivary microbiome profile by cohort based on 16S rRNA gene V3-V4 sequencing. Stacked bar plots depict the sequence abundances of the 15 most abundant genus level taxa in the SLES and HC individuals. The remainder of sequences were combined in the category labeled Other. Taxa were ranked according to the mean abundance across all samples. HCS, HC saliva; SLES, systemic lupus erythematosus saliva.

Supplementary Figure 12 SLES-specific biomarkers. Linear discriminant analysis Effect Size (LEfSe) shows differentially abundant features as biomarkers determined using the Kruskal–Wallis test (*p* < 0.05) with a LDA score > 3.5. Observed features with different abundance in the saliva of patients comparing to the HCs. **(A)** LEfSe cladogram demonstrating microbiome differences at various phylogenic levels; **(B)** LEfSe analysis with linear discriminant analysis (LDA) score representing statistical and biological differences at various phylogenic levels between groups. HCS, HCs saliva; SLES, systemic lupus erythematosus saliva.

Supplementary Figure 13 Comparison of bacterial genus in saliva using SLEDAI score 6 as a cut-off. Statistically significant comparisons after the Wilcoxon rank-sum test and Benjamini–Hochberg false discovery rate (FDR) correction between groups are denoted as *0.05; ** < 0.01; and *** < 0.001. HDAS, high disease activity saliva; HCS, HC saliva; LDAS, low disease activity saliva.

Supplementary Figure 14 Comparison of bacterial genus in saliva when dividing SLE patients into mild, moderate and severe group. Statistically significant comparisons after the Wilcoxon rank-sum test and Benjamini–Hochberg false discovery rate (FDR) correction between groups are denoted as *0.05; ** < 0.01; and *** < 0.001. HCS, HC saliva; MildS, mild saliva; ModerateS, moderate saliva; SevereS, severe saliva.

## Abbreviations

ACE, abundance-based coverage estimators; ActiveF, active feces; ActiveS, active saliva; AHA, anti-histone antibody; ANA, antinuclear antibodies; anti-RNP, Anti-ribonucleoprotein autoantibodies; anti-Sm, anti-Smith antigen antibodies; ANuA, anti-nucleosome antibodies; AR, arthritis; ASV, Amplicon Sequence Variants; C3, complement 3; C4, complement 4; DL, discoid lesions; F/B, Firmicutes/Bacteroidetes; HCF, HC feces; HCS, HC saliva; HD, hematological disorder; InactiveF, inactive feces; InactiveS, inactive saliva; MildF, mild feces; MildS, mild saliva; moderate saliva, ModerateS; ModerateF, moderate feces; MR, malar rash; PCR, Polymerase chain reaction; PH, photosensitivity; principal coordinates analysis, PCoA; QIIME, Quantitative Insights Into Microbial Ecology; RD, renal disorder; RemissiveF, Remissive feces; RemissiveS, remissive saliva; RF, rheumatoid factor; SE, serositis; SevereF, severe feces; SevereS, severe saliva; SLE, systemic lupus erythematosus; SLEDAI, SLE disease activity index; SLEF, SLE feces; SLES, SLE saliva.
