# Supplementary material for: Distinct Microbiomes of Gut and Saliva in Patients With Systemic Lupus Erythematous and Clinical Associations
Source: Front Immunol. 2021 Jul 1;12:626217. doi: 10.3389/fimmu.2021.626217 (PMC8281017; doi:10.3389/fimmu.2021.626217)
Supplement: Supplementary Figure 1 — Bacterial richness and diversity in feces samples of subgroups. (A) Bacterial richness and diversity index compared in fecal samples among LDAF, HDAF and HCF; (B) Bacterial richness and diversity index compared in fecal samples among MildF, ModerateF and SevereF. Statistically significant comparisons after the Wilcoxon rank-sum test and Benjamini–Hochberg false discovery rate (FDR) correction between groups are denoted as *0.05; ** < 0.01; and *** < 0.001. HCF, HC feces; HDAF, High Disease activity feces; LDAF, Low disease activity feces; MildF, mild feces; ModerateF, moderate feces; SevereF, severe feces. [file DataSheet_1.zip › Table S2 Current medication usage.docx]

**Table S2. Current medication usage of SLE patients**

| **ID** | **Medication name** |
| --- | --- |
| SLE1 | Hydroxychloroquine, Prednisone |
| SLE2 | Hydroxychloroquine, Prednisone |
| SLE3 | Hydroxychloroquine, Prednisone |
| SLE4 | Hydroxychloroquine, Prednisone, Leflunomide |
| SLE5 | Hydroxychloroquine, Prednisone, Leflunomide |
| SLE6 | Hydroxychloroquine, Prednisone |
| SLE7 | Hydroxychloroquine, Prednisone |
| SLE8 | Hydroxychloroquine, Prednisone |
| SLE9 | Prednisone, Hydroxychloroquine |
| SLE10 | Prednisone, Hydroxychloroquine |
| SLE11 | Prednisone, Tripterygium wilfordii hook f, Dipyridamole, Leflunomide |
| SLE12 | Hydroxychloroquine, Prednisone |
| SLE13 | Hydroxychloroquine, Prednisone, Leflunomide |
| SLE14 | Hydroxychloroquine, Prednisone |
| SLE15 | Hydroxychloroquine, Prednisone |
| SLE16 | Prednisone |
| SLE17 | Prednisone |
| SLE18 | Hydroxychloroquine, Prednisone |
| SLE19 | Hydroxychloroquine, Leflunomide |
| SLE20 | Prednisone, Hydroxychloroquine |
| SLE21 | Hydroxychloroquine |
| SLE22 | Hydroxychloroquine |
| SLE23 | Hydroxychloroquine, Prednisone |
| SLE24 | Hydroxychloroquine, Prednisone |
| SLE25 | Hydroxychloroquine, Prednisone |
| SLE26 | Hydroxychloroquine, Prednisone |
| SLE27 | Hydroxychloroquine, Leflunomide |
| SLE28 | Hydroxychloroquine, Prednisone, Leflunomide |
| SLE29 | Hydroxychloroquine |
| SLE30 | Hydroxychloroquine, Prednisone, Placenta polypeptide |
| SLE31 | Hydroxychloroquine, Prednisone |
| SLE32 | Hydroxychloroquine, Prednisone |
| SLE33 | Prednisone |
| SLE34 | Hydroxychloroquine, Prednisone |
| SLE35 | Prednisone |
