# Supplementary material for: Distinct Microbiomes of Gut and Saliva in Patients With Systemic Lupus Erythematous and Clinical Associations
Source: Front Immunol. 2021 Jul 1;12:626217. doi: 10.3389/fimmu.2021.626217 (PMC8281017; doi:10.3389/fimmu.2021.626217)
Supplement: Supplementary Figure 1 — Bacterial richness and diversity in feces samples of subgroups. (A) Bacterial richness and diversity index compared in fecal samples among LDAF, HDAF and HCF; (B) Bacterial richness and diversity index compared in fecal samples among MildF, ModerateF and SevereF. Statistically significant comparisons after the Wilcoxon rank-sum test and Benjamini–Hochberg false discovery rate (FDR) correction between groups are denoted as *0.05; ** < 0.01; and *** < 0.001. HCF, HC feces; HDAF, High Disease activity feces; LDAF, Low disease activity feces; MildF, mild feces; ModerateF, moderate feces; SevereF, severe feces. [file DataSheet_1.zip › Table S3 Renal function Parameters in SLE patients.docx]

**Table S3 Renal function Parameters in SLE patients (n = 35)**

| **ID** | **Serum creatinine（35.2-97.5 μmol/L）** | **Blood urea nitrogen（2.1-8.6 mmol/L）** | **Blood uric acid (140-420 μmol/L)** | **Glomerular filtration rate**  **(≥ 90ml/min/1.73m2)** | **Serum albumin (35-55g/L)** | **Proteinuria (Negative)** | **Urinary cellular cast (normal range:002/uL)** |
| --- | --- | --- | --- | --- | --- | --- | --- |
| SLE1 | 45.40 | 4.85 | 321.90 | 118.00 | 41.00 | + + | 0.00 |
| SLE2 | 47.00 | 6.50 | 258.50 | 127.84 | 33.50 | - | 0.12 |
| SLE3 | 59.60 | 4.70 | 330.60 | 124.91 | 33.80 | - | 0.24 |
| SLE4 | 48.70 | 2.77 | 344.30 | 118.00 | 37.10 | - | 0.00 |
| SLE5 | 51.00 | 3.10 | 389.80 | 108.88 | 37.00 | - | 0.00 |
| SLE6 | 53.20 | 4.27 | 471.70 | 90.00 | 33.30 | - | 0.00 |
| SLE7 | 48.16 | 4.35 | 296.53 | 108.88 | 48.40 | - | 0.00 |
| SLE8 | 66.00 | 7.40 | 461.00 | 103.17 | 43.10 | - | 0.12 |
| SLE9 | 44.20 | 3.37 | 306.60 | 114.00 | 41.40 | - | 0.00 |
| SLE10 | 46.30 | 2.99 | 206.80 | 106.15 | 38.80 | - | 0.00 |
| SLE11 | 45.00 | 5.32 | 182.20 | 114.00 | 37.00 | - | 0.00 |
| SLE12 | 61.00 | 5.31 | 172.50 | 103.00 | 39.96 | - | 0.00 |
| SLE13 | 40.60 | 2.37 | 127.70 | 132.00 | 39.20 | + + | 0.00 |
| SLE14 | 54.80 | 6.32 | 322.50 | 108.88 | 33.50 | - | 0.00 |
| SLE15 | 50.00 | 3.80 | 291.10 | 108.88 | 33.80 | - | 1.50 |
| SLE16 | 57.20 | 3.56 | 320.40 | 97.00 | 38.40 | + | 0.00 |
| SLE17 | 55.40 | 5.16 | 271.60 | 103.00 | 46.40 | - | 0.00 |
| SLE18 | 48.16 | 4.35 | 296.53 | 108.88 | 37.70 | - | 0.00 |
| SLE19 | 83.80 | 4.70 | 461.90 | 69.26 | 39.40 | - | 0.00 |
| SLE20 | 50.10 | 2.10 | 346.10 | 123.00 | 29.80 | + + + | 0.00 |
| SLE21 | 35.00 | 4.00 | 248.90 | 108.88 | 42.30 | - | 0.36 |
| SLE22 | 50.00 | 2.70 | 215.60 | 108.88 | 38.00 | - | 0.13 |
| SLE23 | 48.16 | 4.35 | 296.53 | 108.88 | 46.70 | - | 0.00 |
| SLE24 | 48.16 | 4.35 | 296.53 | 108.88 | 50.40 | - | 0.00 |
| SLE25 | 55.80 | 4.66 | 401.60 | 115.00 | 36.00 | + + | 0.00 |
| SLE26 | 48.16 | 4.35 | 296.53 | 108.88 | 39.98 | - | 0.00 |
| SLE27 | 44.60 | 5.67 | 138.40 | 110.00 | 36.20 | - | 0.00 |
| SLE28 | 48.16 | 4.35 | 296.53 | 108.88 | 49.10 | - | 0.00 |
| SLE29 | 45.60 | 4.05 | 275.50 | 129.00 | 43.00 | - | 0.00 |
| SLE30 | 62.70 | 5.30 | 210.70 | 108.88 | 39.97 | - | 0.00 |
| SLE31 | 43.00 | 5.00 | 331.30 | 90.00 | 39.96 | - | 0.00 |
| SLE32 | 66.50 | 4.35 | 342.30 | 95.00 | 36.90 | - | 0.00 |
| SLE33 | 45.90 | 3.47 | 263.20 | 90.00 | 50.80 | - | 0.00 |
| SLE34 | 48.16 | 4.35 | 296.53 | 108.88 | 45.60 | - | 0.00 |
| SLE35 | 43.50 | 3.89 | 288.20 | 123.00 | 41.50 | - | 0.00 |
