# Supplementary material for: Distinct Microbiomes of Gut and Saliva in Patients With Systemic Lupus Erythematous and Clinical Associations
Source: Front Immunol. 2021 Jul 1;12:626217. doi: 10.3389/fimmu.2021.626217 (PMC8281017; doi:10.3389/fimmu.2021.626217)
Supplement: Supplementary Figure 1 — Bacterial richness and diversity in feces samples of subgroups. (A) Bacterial richness and diversity index compared in fecal samples among LDAF, HDAF and HCF; (B) Bacterial richness and diversity index compared in fecal samples among MildF, ModerateF and SevereF. Statistically significant comparisons after the Wilcoxon rank-sum test and Benjamini–Hochberg false discovery rate (FDR) correction between groups are denoted as *0.05; ** < 0.01; and *** < 0.001. HCF, HC feces; HDAF, High Disease activity feces; LDAF, Low disease activity feces; MildF, mild feces; ModerateF, moderate feces; SevereF, severe feces. [file DataSheet_1.zip › Table S7 Comparison of studies on SLE gut microbiome.docx]

**Table S7 Comparison of studies on SLE gut microbiome**

| **Authors** | **Sequence method** | **Subjects of race** | | **Number of subjects** | **Main findings** |
| --- | --- | --- | --- | --- | --- |
| Luo et al[1] | 16S rRNA | American | SLE: 14  CT: 17 | | SLE vs. CT: Shannon index↓; Proteobacteria ↑, f_Rikenellaceae g_ s_↓, *g_Blautia s*_↑, *g_Odoribacter s*_↓ |
| Azzouz D[2] | 16S rRNA | American | SLE: 61  CT: 17 | | 1. SLE vs CT: Chao index↓, *Ruminococcus gnavus*↑ 2. SLEDAI^High^ vs CT: OTU↓, Veillonellaceae↑, Ruminococcaceae↓, *Blautia*↑, *Ruminococcus gnavus*↑, *Bacteroides uniformis*↓ |
| Hevia A[3] | 16S rRNA | Spanish | SLE: 20  CT: 20 | | SLE vs. CT: different β-diversity, F/B ratio↓, *Bacteroides*↑, *Desulfovibrio*↓ |
| He Z[4] | 16S rRNA | Chinese | SLE: 35  CT: 35 | | SLE vs. CT: PD whole tree↓, observed species↓, Singles and Doubles↓, F/B ratio↓, *Rhodococcus*↑, *Eggerthella*↑, *Klebsiella*↑, *Prevotella*↑, *Eubacterium*↑, *Flavonifractor*↑, *Incertae sedis*↑, *Dialister*↓, *Pseudobutyrivibrio*↓ |
| Li Y [5] | 16S rRNA | Chinese | SLE: 40  RA: 20  CT: 22 | | 1. SLE vs. CT: Chao 1↓, Observed species↓, different β-diversity, *Faecalibacterium*↓, *F. prausnitzii*↓, *Cryptophyta*↓, *Roseburia*↓, Streptococcaceae↑, Lactobacillaceae↑, *S. anginosus*↑, *L. mucosae*↑, *Megasphaera*↑ 2. SLE vs. RA: *EB1017*↓, *Ellin6529*↓, *Anaerofilum*↓, Lactobacillales↑, *Streptococcus*↑, *Megasphaera*↑, *Veillonella*↑, *V. dispar*↑ 3. Active vs. Remissive: different β-diversity, *Bifidobacterium*↓, *Ruminococcus. gnavus*↓, Lactobacillales↑, *Streptococcus*↑, *S. anginosus*↑, *Oribacterium*↑, *Epsilonproteobacteria*↑, Campylobacterales↑, *Campylobacter*↑ |
| Rodriguez-Carrio J et al[6] | 16S rRNA | Spanish | SLE: 21  CT: 25 | | 1. SLE vs CT: F/B ratio↓ |
| van der Meulen, T A[7] | 16S rRNA | Netherlands | SLE: 30  pSS: 39  CT: 965 | | SLE vs CT: F/B ratio↓, Chao 1↓, *Bacteroides*↑, *B. vulgatus*↑, *B.uniformis*↑, *B.ovatus*↑, *B. theta*↑, *Clostridium sensu stricto*↓ |
| Wei F[8] | 16S rRNA | Chinese | SLE: 16  CT: 14 | | SLE vs CT: different β-diversity, Proteobacteria↑, Enterobacterlaceae↑, Ruminococcaceae↓, Prevotellaceae↓, XI_o_Clostridiales↓, *Prevotella_9*↓, *Roseburia*↓, Streptococcus↑, *Ruminococcaceae_UCG-003*↓, *Ruminococcaceae_NK4A214_group*↓, *Paraprevotella*↓, *Ruminococcaceae_UCG-013*↓, *Ezakiella*↓, *Porphyromonas*↓ |
| Chen B D[9] | shotgun metagenomic  Sequencing | Chinese | Non-treated SLE: 117  Post-treated SLE: 52  CT: 115 | | 1. Non-treated SLE vs CT: Shannon index↓, *Lactobacillus salivarius*↑, *Clostridium sp. ATCC BAA-442*↑, *Atopobium rimae*↑, *Shuttleworthia satelles*↑, *Actinomyces massiliensis*↑, *Bacteroides fragilis*↑, *Clostridium leptum*↑ 2. Non-treated SLE vs Post-treated SLE: the taxa abovementioned decreased, excluding *L. salivarius* |
| Our present study | 16S rRNA | Chinese | SLE: 35  CT: 35 | | 1. SLE vs CT: bacterial richness and diversity↓, different β-diversity, *Lactobacillus*↑, *L. iners*↑, *f_Ruminococcaceae;g_*↓, *Bifidobacterium*↓, *B. adolescentis*↓, *B. longum*↓, *Prevotella*↑, *Blautia*↑ 2. Subgroups of SLE patients divided by SLEDAI scores: non-significant difference in bacterial richness and diversity, and β-diversity |

Abbreviations: RA, Rheumatoid Arthritis; CT, controls; F / B, Firmicutes / Bacteroidetes

**References**

1. Luo X, Edwards M, Mu Q, Yu Y, Vieson M, Reilly C *et al*. Gut Microbiota in Human Systemic Lupus Erythematosus and a Mouse Model of Lupus. *Appl Environ Microbiol* (2018) **84**: e2217-e2288.

2. Azzouz D, Omarbekova A, Heguy A, Schwudke D, Gisch N, Rovin BH *et al*. Lupus nephritis is linked to disease-activity associated expansions and immunity to a gut commensal. *ANN RHEUM DIS* (2019) **78**: 947-956.

3. Hevia A, Milani C, Lopez P, Cuervo A, Arboleya S, Duranti S *et al*. Intestinal dysbiosis associated with systemic lupus erythematosus. *MBIO* (2014) **5**: e1514-e1548.

4. He Z, Shao T, Li H, Xie Z, Wen C. Alterations of the gut microbiome in Chinese patients with systemic lupus erythematosus. *GUT PATHOG* (2016) **8**: 64.

5. Li Y, Wang H, Li X, Li H, Zhang Q, Zhou H *et al*. Disordered intestinal microbes are associated with the activity of Systemic Lupus Erythematosus. *CLIN SCI* (2019) **133**: 821-838.

6. Rodriguez-Carrio J, Lopez P, Sanchez B, Gonzalez S, Gueimonde M, Margolles A *et al*. Intestinal Dysbiosis Is Associated with Altered Short-Chain Fatty Acids and Serum-Free Fatty Acids in Systemic Lupus Erythematosus. *FRONT IMMUNOL* (2017) **8**: 23.

7. van der Meulen TA, Harmsen H, Vila AV, Kurilshikov A, Liefers SC, Zhernakova A *et al*. Shared gut, but distinct oral microbiota composition in primary Sjogren's syndrome and systemic lupus erythematosus. *J AUTOIMMUN* (2019) **97**: 77-87.

8. Wei F, Xu H, Yan C, Rong C, Liu B, Zhou H. Changes of intestinal flora in patients with systemic lupus erythematosus in northeast China. *PLOS ONE* (2019) **14**: e213063.

9. Chen BD, Jia XM, Xu JY, Zhao LD, Ji JY, Wu BX *et al*. An Autoimmunogenic and Proinflammatory Profile Defined by the Gut Microbiota of Patients With Untreated Systemic Lupus Erythematosus. *ARTHRITIS RHEUMATOL* (2021) **73**: 232-243.
