# Supplementary material for: Distinct Microbiomes of Gut and Saliva in Patients With Systemic Lupus Erythematous and Clinical Associations
Source: Front Immunol. 2021 Jul 1;12:626217. doi: 10.3389/fimmu.2021.626217 (PMC8281017; doi:10.3389/fimmu.2021.626217)
Supplement: Supplementary Figure 1 — Bacterial richness and diversity in feces samples of subgroups. (A) Bacterial richness and diversity index compared in fecal samples among LDAF, HDAF and HCF; (B) Bacterial richness and diversity index compared in fecal samples among MildF, ModerateF and SevereF. Statistically significant comparisons after the Wilcoxon rank-sum test and Benjamini–Hochberg false discovery rate (FDR) correction between groups are denoted as *0.05; ** < 0.01; and *** < 0.001. HCF, HC feces; HDAF, High Disease activity feces; LDAF, Low disease activity feces; MildF, mild feces; ModerateF, moderate feces; SevereF, severe feces. [file DataSheet_1.zip › Table S1 Characteristics of participants.doc]

**Table S1.** Characteristics of patients and controls

| Characteristic | Value for corhort (n^a^)^b^ or statistic | | *p* value^c^ |
| --- | --- | --- | --- |
|  | SLE (n = 35) | HCs (n = 35) |  |
| Female sex, n (%) | 3 (8.57) | 3 (8.57) | 1.000 |
| Age | 41.46 ± 12.36 | 45.06 ± 11.24 | 0.207 |
| Body-mass index (kg/m^2^) | 23.03 ± 3.57 | 24.56 ± 2.89 | 0.053 |
| Smoking, n (%) | 0 (0.00) | 0 (0.00) | 1.000 |
| Drinking, n (%) | 0 (0.00) | 0 (0.00) | 1.000 |
| Type 2 diabetic mellitus, n (%) | 2 (5.71) | 4 (11.43) | 0.421 |
| Hypertension, n (%) | 2 (5.71) | 4 (11.43) | 0.421 |
| Complement C3 (g/L) | 0.81 ± 0.23 | 1.03 ± 0.12 | 0.000 |
| Complement C4 (g/L) | 0.15 ± 0.06 | 0.19 ± 0.02 | 0.000 |

^a^ n, number of subjects.;

^b^ Mean ± SD or n (%)

^c^ Fisher’s exact tests were used with categorical variables. Student’s *t* test with continuous variables.
