# Supplementary material for: Distinct Microbiomes of Gut and Saliva in Patients With Systemic Lupus Erythematous and Clinical Associations
Source: Front Immunol. 2021 Jul 1;12:626217. doi: 10.3389/fimmu.2021.626217 (PMC8281017; doi:10.3389/fimmu.2021.626217)
Supplement: Supplementary Figure 1 — Bacterial richness and diversity in feces samples of subgroups. (A) Bacterial richness and diversity index compared in fecal samples among LDAF, HDAF and HCF; (B) Bacterial richness and diversity index compared in fecal samples among MildF, ModerateF and SevereF. Statistically significant comparisons after the Wilcoxon rank-sum test and Benjamini–Hochberg false discovery rate (FDR) correction between groups are denoted as *0.05; ** < 0.01; and *** < 0.001. HCF, HC feces; HDAF, High Disease activity feces; LDAF, Low disease activity feces; MildF, mild feces; ModerateF, moderate feces; SevereF, severe feces. [file DataSheet_1.zip › Table S4 Sequence in negative control samples constructed from biom file.doc]

**Table S4. Sequence in negative control samples constructed from biom file**

| **ASVs ID** | **NSC1** | **NSC2** | **NSC3** | **NTC1** | **NTC2** | **NTC3** | **NTC4** | **NTC5** | **NTC6** | **Taxonomy** | |
| --- | --- | --- | --- | --- | --- | --- | --- | --- | --- | --- | --- |
| 994 | 4 | 7 | 2 | 0 | 0 | 0 | 0 | 0 | 0 | k__Bacteria; p__Proteobacteria; c__Gammaproteobacteria; o__Enterobacteriales; f__Enterobacteriaceae; g__Providencia; s__ |  |
| 3765 | 2 | 1 | 0 | 0 | 0 | 0 | 0 | 0 | 0 | k__Bacteria; p__Proteobacteria; c__Betaproteobacteria; o__Burkholderiales; f__Alcaligenaceae; g__Alcaligenes; s__faecalis |  |
| 25 | 2 | 0 | 0 | 0 | 0 | 0 | 0 | 0 | 0 | k__Bacteria; p__Cyanobacteria; c__Chloroplast; o__Streptophyta; f__; g__; s__ |  |
| 2200 | 1 | 5 | 2 | 0 | 0 | 0 | 0 | 0 | 0 | k__Bacteria; p__Proteobacteria; c__Gammaproteobacteria; o__Enterobacteriales; f__Enterobacteriaceae; g__; s__ |  |
| 979 | 1 | 1 | 1 | 0 | 0 | 0 | 0 | 0 | 0 | k__Bacteria; p__Proteobacteria; c__Gammaproteobacteria; o__Enterobacteriales; f__Enterobacteriaceae; g__Providencia; s__ |  |
| 1812 | 1 | 0 | 0 | 0 | 0 | 0 | 0 | 0 | 0 | k__Bacteria; p__Proteobacteria; c__Gammaproteobacteria; o__Enterobacteriales; f__Enterobacteriaceae; g__Providencia; s__ |  |
| 259 | 0 | 0 | 0 | 0 | 3 | 0 | 0 | 0 | 7 | k__Bacteria; p__Proteobacteria; c__Gammaproteobacteria; o__Xanthomonadales; f__Xanthomonadaceae |  |
| 113 | 0 | 0 | 0 | 0 | 5 | 0 | 135 | 6 | 4 | k__Bacteria; p__Firmicutes; c__Bacilli; o__Bacillales; f__Planococcaceae; g__Lysinibacillus; s__boronitolerans |  |
| 422 | 0 | 0 | 0 | 0 | 6 | 0 | 0 | 0 | 3 | k__Bacteria; p__Firmicutes; c__Clostridia; o__MBA08; f__; g__; s__ |  |
| 381 | 0 | 0 | 0 | 4 | 3 | 0 | 0 | 0 | 2 | k__Bacteria; p__Firmicutes; c__Clostridia; o__MBA08; f__; g__; s__ |  |
| 383 | 0 | 0 | 0 | 0 | 1 | 0 | 0 | 0 | 2 | k__Bacteria; p__Proteobacteria; c__Gammaproteobacteria; o__Pseudomonadales; f__Pseudomonadaceae; g__; s__ |  |
| 6 | 0 | 0 | 1 | 2 | 52 | 6 | 450 | 47 | 1 | k__Bacteria; p__Proteobacteria; c__Gammaproteobacteria; o__Enterobacteriales; f__Enterobacteriaceae; g__; s__ |  |
| 118 | 0 | 0 | 0 | 1 | 4 | 0 | 14 | 0 | 1 | k__Bacteria; p__Proteobacteria; c__Betaproteobacteria; o__Burkholderiales; f__Oxalobacteraceae; g__Ralstonia; s__ |  |
| 98 | 0 | 0 | 0 | 0 | 2 | 0 | 28 | 15 | 1 | k__Bacteria; p__Firmicutes; c__Bacilli; o__Lactobacillales; f__Streptococcaceae; g__Streptococcus; s__ |  |
| 166 | 0 | 0 | 0 | 0 | 1 | 0 | 0 | 2 | 1 | k__Bacteria; p__Firmicutes; c__Clostridia; o__Clostridiales; f__Veillonellaceae; g__Veillonella; s__dispar |  |
| 19 | 0 | 0 | 0 | 2 | 0 | 0 | 0 | 1 | 1 | k__Bacteria; p__Firmicutes; c__Clostridia; o__Clostridiales; f__Ruminococcaceae; g__; s__ |  |
| 223 | 0 | 0 | 0 | 0 | 0 | 0 | 0 | 0 | 1 | k__Bacteria; p__Firmicutes; c__Clostridia; o__Clostridiales; f__Veillonellaceae; g__Megamonas; s__ |  |
| 11 | 0 | 0 | 0 | 0 | 0 | 0 | 0 | 0 | 1 | k__Bacteria; p__Firmicutes; c__Clostridia; o__Clostridiales; f__Veillonellaceae; g__Megamonas; s__ |  |
| 725 | 0 | 0 | 0 | 0 | 0 | 0 | 0 | 0 | 1 | k__Bacteria; p__Bacteroidetes; c__Bacteroidia; o__Bacteroidales; f__Bacteroidaceae; g__Bacteroides; s__caccae |  |
| 7 | 0 | 0 | 0 | 0 | 0 | 0 | 0 | 0 | 1 | k__Bacteria; p__Firmicutes; c__Clostridia; o__Clostridiales; f__Veillonellaceae; g__Veillonella; s__dispar |  |
| 33 | 0 | 0 | 0 | 0 | 0 | 0 | 0 | 0 | 1 | k__Bacteria; p__Actinobacteria; c__Actinobacteria; o__Bifidobacteriales; f__Bifidobacteriaceae; g__Bifidobacterium; s__longum |  |
| 9 | 0 | 0 | 0 | 0 | 0 | 0 | 0 | 0 | 1 | k__Bacteria; p__Bacteroidetes; c__Bacteroidia; o__Bacteroidales; f__Bacteroidaceae; g__Bacteroides; s__uniformis |  |
| 201 | 0 | 0 | 0 | 0 | 0 | 0 | 0 | 0 | 1 | k__Bacteria; p__Fusobacteria; c__Fusobacteriia; o__Fusobacteriales; f__Leptotrichiaceae; g__Leptotrichia; s__ |  |
| 80 | 0 | 0 | 0 | 0 | 0 | 0 | 0 | 0 | 1 | k__Bacteria; p__Firmicutes; c__Clostridia; o__Clostridiales; f__Veillonellaceae; g__Megamonas; s__ |  |
| 26 | 0 | 0 | 0 | 0 | 1 | 2 | 1 | 3 | 0 | k__Bacteria; p__Firmicutes; c__Bacilli; o__Lactobacillales; f__Streptococcaceae; g__Streptococcus; s__ |  |
| 1664 | 0 | 0 | 0 | 0 | 0 | 1 | 0 | 0 | 0 | k__Bacteria; p__Bacteroidetes; c__Bacteroidia; o__Bacteroidales; f__Porphyromonadaceae; g__Parabacteroides; s__ |  |
| 47 | 0 | 0 | 0 | 0 | 0 | 1 | 1 | 0 | 0 | k__Bacteria; p__Actinobacteria; c__Actinobacteria; o__Actinomycetales; f__Micrococcaceae; g__Rothia; s__mucilaginosa |  |
| 2383 | 0 | 0 | 0 | 0 | 0 | 1 | 0 | 0 | 0 | k__Bacteria; p__Proteobacteria; c__Alphaproteobacteria; o__Rhodobacterales; f__Rhodobacteraceae; g__Paracoccus; s__ |  |
| 468 | 0 | 0 | 0 | 0 | 0 | 1 | 0 | 0 | 0 | k__Bacteria; p__Spirochaetes; c__Spirochaetes; o__Spirochaetales; f__Spirochaetaceae; g__Treponema; s__ |  |
| 21 | 0 | 0 | 0 | 0 | 0 | 1 | 1 | 0 | 0 | k__Bacteria; p__Proteobacteria; c__Betaproteobacteria; o__Neisseriales; f__Neisseriaceae; g__Neisseria; s__cinerea |  |
| 706 | 0 | 0 | 0 | 0 | 0 | 1 | 0 | 0 | 0 | k__Bacteria; p__Bacteroidetes; c__Bacteroidia; o__Bacteroidales; f__Prevotellaceae; g__Prevotella; s__ |  |
| 773 | 0 | 0 | 0 | 0 | 0 | 1 | 0 | 0 | 0 | k__Bacteria; p__Firmicutes; c__Clostridia; o__Clostridiales; f__Veillonellaceae; g__Megasphaera; s__ |  |
| 182 | 0 | 0 | 0 | 0 | 0 | 1 | 0 | 0 | 0 | k__Bacteria; p__Firmicutes; c__Clostridia; o__Clostridiales; f__Lachnospiraceae; g__Anaerostipes; s__ |  |
| 934 | 0 | 0 | 0 | 0 | 7 | 0 | 0 | 0 | 0 | k__Bacteria; p__Firmicutes; c__Bacilli; o__Bacillales; f__; g__; s__ |  |
| 3528 | 0 | 0 | 0 | 0 | 7 | 0 | 0 | 0 | 0 | k__Bacteria; p__Firmicutes; c__Clostridia; o__Clostridiales; f__[Tissierellaceae]; g__Tepidimicrobium; s__ |  |
| 1096 | 0 | 0 | 0 | 0 | 6 | 0 | 0 | 0 | 0 | k__Bacteria; p__Bacteroidetes; c__Bacteroidia; o__Bacteroidales; f__Marinilabiaceae; g__; s__ |  |
| 804 | 0 | 0 | 0 | 0 | 4 | 0 | 0 | 0 | 0 | k__Bacteria; p__Firmicutes; c__Bacilli; o__Bacillales; f__Bacillaceae; g__Bacillus; s__ |  |
| 715 | 0 | 0 | 0 | 0 | 3 | 0 | 1 | 0 | 0 | k__Bacteria; p__Proteobacteria; c__Betaproteobacteria; o__Rhodocyclales; f__Rhodocyclaceae; g__Hydrogenophilus; s__ |  |
| 964 | 0 | 0 | 0 | 0 | 2 | 0 | 0 | 0 | 0 | k__Bacteria; p__Firmicutes; c__Clostridia; o__Clostridiales; f__Caldicoprobacteraceae; g__Caldicoprobacter; s__ |  |
| 169 | 0 | 0 | 0 | 0 | 2 | 0 | 20 | 0 | 0 | k__Bacteria; p__Proteobacteria; c__Alphaproteobacteria; o__Rhizobiales; f__Brucellaceae; g__Ochrobactrum; s__ |  |
| 469 | 0 | 0 | 0 | 0 | 1 | 0 | 1 | 0 | 0 | k__Bacteria; p__Proteobacteria; c__Gammaproteobacteria; o__Pseudomonadales; f__Moraxellaceae; g__Acinetobacter; s__johnsonii |  |
| 62 | 0 | 0 | 0 | 0 | 1 | 0 | 0 | 0 | 0 | k__Bacteria; p__Firmicutes; c__Clostridia; o__Clostridiales; f__Ruminococcaceae; g__; s__ |  |
| 140 | 0 | 0 | 0 | 0 | 1 | 0 | 0 | 0 | 0 | k__Bacteria; p__Proteobacteria; c__Alphaproteobacteria; o__Sphingomonadales; f__Sphingomonadaceae; g__Sphingomonas; s__yabuuchiae |  |
| 1856 | 0 | 0 | 0 | 0 | 1 | 0 | 0 | 0 | 0 | k__Bacteria; p__Firmicutes; c__Clostridia; o__Clostridiales; f__Ruminococcaceae; g__Ruminococcus; s__bromii |  |
| 4156 | 0 | 0 | 0 | 0 | 1 | 0 | 0 | 0 | 0 | k__Bacteria; p__Firmicutes; c__Bacilli; o__Bacillales; f__[Exiguobacteraceae]; g__Exiguobacterium; s__ |  |
| 2657 | 0 | 0 | 0 | 0 | 1 | 0 | 0 | 0 | 0 | k__Bacteria; p__Firmicutes; c__Bacilli; o__Bacillales; f__Bacillaceae; g__Bacillus; s__ |  |
| 3228 | 0 | 0 | 0 | 0 | 1 | 0 | 0 | 0 | 0 | k__Bacteria; p__Bacteroidetes; c__Bacteroidia; o__Bacteroidales; f__Marinilabiaceae; g__; s__ |  |
| 471 | 0 | 0 | 0 | 11 | 0 | 0 | 0 | 0 | 0 | k__Bacteria; p__Bacteroidetes; c__Bacteroidia; o__Bacteroidales; f__S24-7; g__; s__ |  |
| 363 | 0 | 0 | 0 | 10 | 0 | 0 | 0 | 0 | 0 | k__Bacteria; p__Proteobacteria; c__Betaproteobacteria; o__Burkholderiales; f__Alcaligenaceae; g__Sutterella; s__ |  |
| 339 | 0 | 0 | 0 | 10 | 0 | 0 | 0 | 0 | 0 | k__Bacteria; p__Bacteroidetes; c__Bacteroidia; o__Bacteroidales; f__S24-7; g__; s__ |  |
| 89 | 0 | 0 | 0 | 9 | 0 | 0 | 0 | 0 | 0 | k__Bacteria; p__Bacteroidetes; c__Bacteroidia; o__Bacteroidales; f__S24-7; g__; s__ |  |
| 94 | 0 | 0 | 3 | 7 | 0 | 0 | 0 | 0 | 0 | k__Bacteria; p__Bacteroidetes; c__Bacteroidia; o__Bacteroidales; f__S24-7; g__; s__ |  |
| 385 | 0 | 0 | 0 | 7 | 0 | 0 | 0 | 0 | 0 | k__Bacteria; p__Proteobacteria; c__Betaproteobacteria; o__Neisseriales; f__Neisseriaceae; g__Neisseria; s__subflava |  |
| 202 | 0 | 0 | 0 | 6 | 0 | 0 | 0 | 0 | 0 | k__Bacteria; p__Bacteroidetes; c__Bacteroidia; o__Bacteroidales; f__S24-7; g__; s__ |  |
| 1265 | 0 | 0 | 0 | 6 | 0 | 0 | 0 | 0 | 0 | k__Bacteria; p__Bacteroidetes; c__Bacteroidia; o__Bacteroidales; f__S24-7; g__; s__ |  |
| 2306 | 0 | 0 | 0 | 5 | 0 | 0 | 0 | 0 | 0 | k__Bacteria; p__Bacteroidetes; c__Bacteroidia; o__Bacteroidales; f__S24-7; g__; s__ |  |
| 1585 | 0 | 0 | 0 | 3 | 0 | 0 | 0 | 0 | 0 | k__Bacteria; p__Bacteroidetes; c__Bacteroidia; o__Bacteroidales; f__S24-7; g__; s__ |  |
| 1533 | 0 | 0 | 0 | 3 | 0 | 0 | 0 | 0 | 0 | k__Bacteria; p__Bacteroidetes; c__Bacteroidia; o__Bacteroidales; f__S24-7; g__; s__ |  |
| 980 | 0 | 0 | 0 | 3 | 0 | 0 | 0 | 0 | 0 | k__Bacteria; p__Bacteroidetes; c__Bacteroidia; o__Bacteroidales; f__S24-7; g__; s__ |  |
| 522 | 0 | 0 | 0 | 3 | 0 | 0 | 0 | 0 | 0 | k__Bacteria; p__Bacteroidetes; c__Bacteroidia; o__Bacteroidales; f__S24-7; g__; s__ |  |
| 675 | 0 | 0 | 0 | 3 | 0 | 0 | 0 | 0 | 0 | k__Bacteria; p__Bacteroidetes; c__Bacteroidia; o__Bacteroidales; f__S24-7; g__; s__ |  |
| 1019 | 0 | 0 | 0 | 3 | 0 | 0 | 0 | 0 | 0 | k__Bacteria; p__Bacteroidetes; c__Bacteroidia; o__Bacteroidales; f__S24-7; g__; s__ |  |
| 396 | 0 | 0 | 0 | 2 | 0 | 0 | 0 | 0 | 0 | k__Bacteria; p__Bacteroidetes; c__Bacteroidia; o__Bacteroidales; f__S24-7; g__; s__ |  |
| 268 | 0 | 0 | 0 | 2 | 0 | 0 | 0 | 0 | 0 | k__Bacteria; p__Firmicutes; c__Clostridia; o__Clostridiales; f__Veillonellaceae; g__Dialister; s__ |  |
| 199 | 0 | 0 | 0 | 2 | 0 | 0 | 0 | 0 | 0 | k__Bacteria; p__Bacteroidetes; c__Bacteroidia; o__Bacteroidales; f__S24-7; g__; s__ |  |
| 1886 | 0 | 0 | 0 | 2 | 0 | 0 | 0 | 0 | 0 | k__Bacteria; p__Firmicutes; c__Clostridia; o__Clostridiales; f__; g__; s__ |  |
| 3477 | 0 | 0 | 0 | 2 | 0 | 0 | 0 | 0 | 0 | k__Bacteria; p__Firmicutes; c__Clostridia; o__Clostridiales; f__Ruminococcaceae; g__Oscillospira; s__ |  |
| 332 | 0 | 0 | 0 | 2 | 0 | 0 | 0 | 0 | 0 | k__Bacteria; p__Bacteroidetes; c__Bacteroidia; o__Bacteroidales; f__S24-7; g__; s__ |  |
| 748 | 0 | 0 | 0 | 2 | 0 | 0 | 0 | 1 | 0 | k__Bacteria; p__Firmicutes; c__Clostridia; o__Clostridiales; f__Veillonellaceae; g__Veillonella; s__dispar |  |
| 3 | 0 | 0 | 0 | 2 | 0 | 0 | 0 | 0 | 0 | k__Bacteria; p__Proteobacteria; c__Gammaproteobacteria; o__Pasteurellales; f__Pasteurellaceae; g__Haemophilus; s__parainfluenzae |  |
| 13 | 0 | 0 | 0 | 1 | 0 | 0 | 0 | 0 | 0 | k__Bacteria; p__Bacteroidetes; c__Bacteroidia; o__Bacteroidales; f__Prevotellaceae; g__Prevotella; s__nanceiensis |  |
| 107 | 0 | 0 | 0 | 1 | 0 | 0 | 0 | 0 | 0 | k__Bacteria; p__Firmicutes; c__Clostridia; o__Clostridiales; f__Ruminococcaceae; g__; s__ |  |
| 631 | 0 | 0 | 0 | 1 | 0 | 0 | 0 | 0 | 0 | k__Bacteria; p__Bacteroidetes; c__Bacteroidia; o__Bacteroidales; f__S24-7; g__; s__ |  |
| 251 | 0 | 0 | 0 | 1 | 0 | 0 | 0 | 0 | 0 | k__Bacteria; p__Proteobacteria; c__Betaproteobacteria; o__Neisseriales; f__Neisseriaceae; g__Neisseria |  |
| 618 | 0 | 0 | 0 | 1 | 0 | 0 | 0 | 0 | 0 | k__Bacteria; p__Proteobacteria; c__Gammaproteobacteria; o__Enterobacteriales; f__Enterobacteriaceae; g__; s__ |  |
| 328 | 0 | 0 | 0 | 1 | 0 | 0 | 0 | 0 | 0 | k__Bacteria; p__Proteobacteria; c__Gammaproteobacteria; o__Xanthomonadales; f__Xanthomonadaceae |  |
| 542 | 0 | 0 | 0 | 1 | 0 | 0 | 0 | 0 | 0 | k__Bacteria; p__Firmicutes; c__Clostridia; o__Clostridiales; f__Lachnospiraceae; g__Coprococcus; s__eutactus |  |
| 991 | 0 | 0 | 0 | 1 | 0 | 0 | 0 | 0 | 0 | k__Bacteria; p__Firmicutes; c__Erysipelotrichi; o__Erysipelotrichales; f__Erysipelotrichaceae; g__Bulleidia; s__ |  |
| 65 | 0 | 0 | 0 | 1 | 0 | 0 | 0 | 0 | 0 | k__Bacteria; p__Firmicutes; c__Clostridia; o__Clostridiales; f__Lachnospiraceae; g__Blautia; s__ |  |
| 350 | 0 | 0 | 0 | 1 | 0 | 0 | 0 | 0 | 0 | k__Bacteria; p__Bacteroidetes; c__Bacteroidia; o__Bacteroidales; f__S24-7; g__; s__ |  |
| 4118 | 0 | 0 | 0 | 1 | 0 | 0 | 0 | 0 | 0 | k__Bacteria; p__Bacteroidetes; c__Bacteroidia; o__Bacteroidales; f__S24-7; g__; s__ |  |
| 209 | 0 | 0 | 0 | 1 | 0 | 0 | 0 | 0 | 0 | k__Bacteria; p__Bacteroidetes; c__Bacteroidia; o__Bacteroidales; f__S24-7; g__; s__ |  |
| 207 | 0 | 0 | 0 | 1 | 0 | 0 | 0 | 0 | 0 | k__Bacteria; p__Fusobacteria; c__Fusobacteriia; o__Fusobacteriales; f__Leptotrichiaceae; g__Leptotrichia; s__ |  |
| 2006 | 0 | 0 | 0 | 1 | 0 | 0 | 0 | 0 | 0 | k__Bacteria; p__Bacteroidetes; c__Bacteroidia; o__Bacteroidales; f__S24-7; g__; s__ |  |
| 395 | 0 | 0 | 0 | 1 | 0 | 0 | 0 | 0 | 0 | k__Bacteria; p__Bacteroidetes; c__Bacteroidia; o__Bacteroidales; f__S24-7; g__; s__ |  |
| 341 | 0 | 0 | 0 | 1 | 0 | 0 | 0 | 0 | 0 | k__Bacteria; p__Actinobacteria; c__Actinobacteria; o__Bifidobacteriales; f__Bifidobacteriaceae; g__Bifidobacterium; s__pseudolongum |  |
| 143 | 0 | 0 | 0 | 1 | 0 | 0 | 0 | 0 | 0 | k__Bacteria; p__Bacteroidetes; c__Bacteroidia; o__Bacteroidales; f__[Paraprevotellaceae]; g__[Prevotella]; s__ |  |
| 1916 | 0 | 1 | 16 | 0 | 0 | 0 | 0 | 0 | 0 | k__Bacteria; p__Proteobacteria; c__Gammaproteobacteria; o__Pseudomonadales; f__Pseudomonadaceae; g__Pseudomonas; s__ |  |
| 5 | 0 | 1 | 4 | 0 | 0 | 0 | 0 | 0 | 0 | k__Bacteria; p__Firmicutes; c__Bacilli; o__Bacillales; f__Staphylococcaceae; g__Staphylococcus; s__aureus |  |
| 666 | 0 | 0 | 4 | 0 | 0 | 0 | 0 | 0 | 0 | k__Bacteria; p__Bacteroidetes; c__Bacteroidia; o__Bacteroidales; f__S24-7; g__; s__ |  |
| 2858 | 0 | 1 | 3 | 0 | 0 | 0 | 0 | 0 | 0 | k__Bacteria; p__Proteobacteria; c__Alphaproteobacteria; o__Rhizobiales; f__Brucellaceae |  |
| 2420 | 0 | 0 | 3 | 0 | 0 | 0 | 0 | 0 | 0 | k__Bacteria; p__Bacteroidetes; c__Bacteroidia; o__Bacteroidales; f__S24-7; g__; s__ |  |
| 382 | 0 | 0 | 2 | 0 | 0 | 0 | 0 | 0 | 0 | k__Bacteria; p__Proteobacteria; c__Gammaproteobacteria; o__Alteromonadales; f__Shewanellaceae; g__Shewanella; s__algae |  |
| 2531 | 0 | 0 | 2 | 0 | 0 | 0 | 0 | 0 | 0 | k__Bacteria; p__Proteobacteria; c__Epsilonproteobacteria; o__Campylobacterales; f__Helicobacteraceae; g__; s__ |  |
| 1364 | 0 | 0 | 2 | 0 | 0 | 0 | 0 | 0 | 0 | k__Bacteria; p__Proteobacteria; c__Gammaproteobacteria; o__Pseudomonadales; f__Pseudomonadaceae; g__Pseudomonas; s__ |  |
| 692 | 0 | 0 | 1 | 0 | 0 | 0 | 0 | 0 | 0 | k__Bacteria; p__Bacteroidetes; c__Bacteroidia; o__Bacteroidales; f__S24-7; g__; s__ |  |
| 1867 | 0 | 0 | 1 | 0 | 0 | 0 | 0 | 0 | 0 | k__Bacteria; p__Firmicutes; c__Bacilli; o__Bacillales; f__Bacillaceae; g__Anoxybacillus; s__kestanbolensis |  |
| 4503 | 0 | 0 | 1 | 0 | 0 | 0 | 0 | 0 | 0 | k__Bacteria; p__Actinobacteria; c__Actinobacteria; o__Actinomycetales; f__Nocardioidaceae; g__; s__ |  |
| 3838 | 0 | 0 | 1 | 0 | 0 | 0 | 0 | 0 | 0 | k__Bacteria; p__Proteobacteria; c__Alphaproteobacteria; o__Rhodobacterales; f__Rhodobacteraceae; g__Paracoccus |  |
| 3367 | 0 | 0 | 1 | 0 | 0 | 0 | 0 | 0 | 0 | k__Bacteria; p__Proteobacteria; c__Betaproteobacteria; o__Burkholderiales; f__Comamonadaceae; g__; s__ |  |
| 3268 | 0 | 0 | 1 | 0 | 0 | 0 | 0 | 0 | 0 | k__Bacteria; p__Proteobacteria; c__Gammaproteobacteria; o__Pseudomonadales; f__Pseudomonadaceae; g__Pseudomonas; s__ |  |
| 3541 | 0 | 2 | 0 | 0 | 0 | 0 | 0 | 0 | 0 | k__Bacteria; p__Bacteroidetes; c__Flavobacteriia; o__Flavobacteriales; f__Flavobacteriaceae; g__Myroides; s__odoratimimus |  |
| 1983 | 0 | 1 | 0 | 0 | 0 | 0 | 0 | 0 | 0 | k__Bacteria; p__Acidobacteria; c__Acidobacteria-6; o__iii1-15; f__mb2424; g__; s__ |  |
| 158 | 0 | 0 | 0 | 0 | 0 | 0 | 0 | 1 | 0 | k__Bacteria; p__Firmicutes; c__Clostridia; o__Clostridiales; f__Clostridiaceae; g__Clostridium; s__ |  |
| 562 | 0 | 0 | 0 | 0 | 0 | 0 | 1 | 0 | 0 | k__Bacteria; p__Actinobacteria; c__Actinobacteria; o__Actinomycetales; f__Actinomycetaceae; g__Actinomyces; s__ |  |
| 1340 | 0 | 0 | 0 | 0 | 0 | 0 | 4 | 0 | 0 | k__Bacteria; p__Bacteroidetes; c__Bacteroidia; o__Bacteroidales; f__Rikenellaceae; g__; s__ |  |
| 1735 | 0 | 0 | 0 | 0 | 0 | 0 | 3 | 0 | 0 | k__Bacteria; p__Proteobacteria; c__Gammaproteobacteria; o__Pseudomonadales; f__Moraxellaceae; g__Acinetobacter; s__schindleri |  |
| 432 | 0 | 0 | 0 | 0 | 0 | 0 | 0 | 2 | 0 | k__Bacteria; p__Proteobacteria; c__Betaproteobacteria; o__Burkholderiales; f__Burkholderiaceae; g__Lautropia; s__ |  |
| 1559 | 0 | 0 | 0 | 0 | 0 | 0 | 19 | 0 | 0 | k__Bacteria; p__Proteobacteria; c__Alphaproteobacteria; o__Caulobacterales; f__Caulobacteraceae; g__; s__ |  |
| 2049 | 0 | 0 | 0 | 0 | 0 | 0 | 0 | 1 | 0 | k__Bacteria; p__Firmicutes; c__Clostridia; o__Clostridiales; f__Veillonellaceae; g__Veillonella; s__dispar |  |
| 18 | 0 | 0 | 0 | 0 | 0 | 0 | 0 | 1 | 0 | k__Bacteria; p__Actinobacteria; c__Coriobacteriia; o__Coriobacteriales; f__Coriobacteriaceae; g__; s__ |  |
| 28 | 0 | 0 | 0 | 0 | 0 | 0 | 0 | 2 | 0 | k__Bacteria; p__Proteobacteria; c__Betaproteobacteria; o__Neisseriales; f__Neisseriaceae; g__Neisseria; s__ |  |
| 521 | 0 | 0 | 0 | 0 | 0 | 0 | 1 | 1 | 0 | k__Bacteria; p__Firmicutes; c__Bacilli; o__Lactobacillales; f__Lactobacillaceae; g__Lactobacillus; s__ |  |
| 1204 | 0 | 0 | 0 | 0 | 0 | 0 | 1 | 0 | 0 | k__Bacteria; p__Proteobacteria; c__Betaproteobacteria; o__Burkholderiales; f__Alcaligenaceae; g__Achromobacter; s__ |  |
| 120 | 0 | 0 | 0 | 0 | 0 | 0 | 0 | 2 | 0 | k__Bacteria; p__Firmicutes; c__Bacilli; o__Lactobacillales; f__Streptococcaceae; g__Streptococcus; s__ |  |
| 2844 | 0 | 0 | 0 | 0 | 0 | 0 | 10 | 0 | 0 | k__Bacteria; p__Proteobacteria; c__Gammaproteobacteria; o__Enterobacteriales; f__Enterobacteriaceae; g__Erwinia |  |
| 2462 | 0 | 0 | 0 | 0 | 0 | 0 | 2 | 0 | 0 | k__Bacteria; p__Proteobacteria; c__Betaproteobacteria; o__Burkholderiales; f__Comamonadaceae; g__Ramlibacter; s__ |  |
| 23 | 0 | 0 | 0 | 0 | 0 | 0 | 0 | 1 | 0 | k__Bacteria; p__Actinobacteria; c__Actinobacteria; o__Bifidobacteriales; f__Bifidobacteriaceae |  |
| 999 | 0 | 0 | 0 | 0 | 0 | 0 | 1 | 0 | 0 | k__Bacteria; p__Bacteroidetes; c__Bacteroidia; o__Bacteroidales; f__Prevotellaceae; g__Prevotella; s__ |  |
| 8 | 0 | 0 | 0 | 0 | 0 | 0 | 0 | 3 | 0 | k__Bacteria; p__Actinobacteria; c__Actinobacteria; o__Actinomycetales; f__Micrococcaceae; g__Rothia; s__aeria |  |
| 633 | 0 | 0 | 0 | 0 | 0 | 0 | 0 | 1 | 0 | k__Bacteria; p__Spirochaetes; c__Spirochaetes; o__Spirochaetales; f__Spirochaetaceae; g__Treponema; s__amylovorum |  |
| 789 | 0 | 0 | 0 | 0 | 0 | 0 | 0 | 1 | 0 | k__Bacteria; p__Bacteroidetes; c__Bacteroidia; o__Bacteroidales; f__Prevotellaceae; g__Prevotella; s__ |  |
| 439 | 0 | 0 | 0 | 0 | 0 | 0 | 0 | 1 | 0 | k__Bacteria; p__Firmicutes; c__Clostridia; o__Clostridiales; f__Veillonellaceae; g__Selenomonas; s__noxia |  |
| 820 | 0 | 0 | 0 | 0 | 0 | 0 | 13 | 0 | 0 | k__Bacteria; p__Firmicutes; c__Bacilli; o__Bacillales; f__Bacillaceae; g__Bacillus; s__cereus |  |
| 2960 | 0 | 0 | 0 | 0 | 0 | 0 | 1 | 0 | 0 | k__Bacteria; p__Actinobacteria; c__Coriobacteriia; o__Coriobacteriales; f__Coriobacteriaceae; g__Atopobium; s__ |  |
| 1334 | 0 | 0 | 0 | 0 | 0 | 0 | 0 | 1 | 0 | k__Bacteria; p__Actinobacteria; c__Actinobacteria; o__Actinomycetales; f__Actinomycetaceae; g__; s__ |  |
| 540 | 0 | 0 | 0 | 0 | 0 | 0 | 30 | 0 | 0 | k__Bacteria; p__Actinobacteria; c__Actinobacteria; o__Actinomycetales; f__Propionibacteriaceae; g__Propionibacterium; s__acnes |  |
| 1851 | 0 | 0 | 0 | 0 | 0 | 0 | 0 | 5 | 0 | k__Bacteria; p__Proteobacteria; c__Betaproteobacteria; o__Burkholderiales; f__Comamonadaceae; g__Delftia; s__ |  |
| 1567 | 0 | 0 | 0 | 0 | 0 | 0 | 0 | 1 | 0 | k__Bacteria; p__Bacteroidetes; c__Flavobacteriia; o__Flavobacteriales; f__Flavobacteriaceae; g__Capnocytophaga; s__ |  |
| 2239 | 0 | 0 | 0 | 0 | 0 | 0 | 0 | 1 | 0 | k__Bacteria; p__Actinobacteria; c__Actinobacteria; o__Actinomycetales; f__Actinomycetaceae; g__Actinomyces; s__ |  |
| 2952 | 0 | 0 | 0 | 0 | 0 | 0 | 0 | 1 | 0 | k__Bacteria; p__Proteobacteria; c__Betaproteobacteria; o__Neisseriales; f__Neisseriaceae; g__; s__ |  |
| 281 | 0 | 0 | 0 | 0 | 0 | 0 | 0 | 2 | 0 | k__Bacteria; p__Fusobacteria; c__Fusobacteriia; o__Fusobacteriales; f__Leptotrichiaceae; g__Leptotrichia; s__ |  |
| 153 | 0 | 0 | 0 | 0 | 0 | 0 | 0 | 1 | 0 | k__Bacteria; p__Firmicutes; c__Clostridia; o__Clostridiales; f__; g__; s__ |  |
| 185 | 0 | 0 | 0 | 0 | 0 | 0 | 0 | 1 | 0 | k__Bacteria; p__Firmicutes; c__Clostridia; o__Clostridiales; f__Lachnospiraceae; g__; s__ |  |
| 309 | 0 | 0 | 0 | 0 | 0 | 0 | 0 | 1 | 0 | k__Bacteria; p__Firmicutes; c__Clostridia; o__Clostridiales; f__Veillonellaceae; g__Veillonella; s__dispar |  |
| 130 | 0 | 0 | 0 | 0 | 0 | 0 | 0 | 1 | 0 | k__Bacteria; p__Firmicutes; c__Bacilli; o__Lactobacillales; f__Carnobacteriaceae; g__Granulicatella; s__ |  |
| 1532 | 0 | 0 | 0 | 0 | 0 | 0 | 1 | 0 | 0 | k__Bacteria; p__Proteobacteria; c__Gammaproteobacteria; o__Xanthomonadales; f__Xanthomonadaceae; g__; s__ |  |
| 351 | 0 | 0 | 0 | 0 | 0 | 0 | 0 | 1 | 0 | k__Bacteria; p__Firmicutes; c__Clostridia; o__Clostridiales; f__Veillonellaceae; g__Veillonella; s__dispar |  |
| 22 | 0 | 0 | 0 | 0 | 0 | 0 | 0 | 1 | 0 | k__Bacteria; p__Actinobacteria; c__Actinobacteria; o__Bifidobacteriales; f__Bifidobacteriaceae; g__Bifidobacterium; s__ |  |
| 536 | 0 | 0 | 0 | 0 | 0 | 0 | 0 | 1 | 0 | k__Bacteria; p__Bacteroidetes; c__Bacteroidia; o__Bacteroidales; f__Prevotellaceae; g__Prevotella; s__ |  |
| 2959 | 0 | 0 | 0 | 0 | 0 | 0 | 0 | 2 | 0 | k__Bacteria; p__Proteobacteria; c__Alphaproteobacteria; o__Sphingomonadales; f__Sphingomonadaceae; g__Sphingomonas; s__ |  |
| 155 | 0 | 0 | 0 | 0 | 0 | 0 | 0 | 2 | 0 | k__Bacteria; p__Firmicutes; c__Clostridia; o__Clostridiales; f__Veillonellaceae; g__Veillonella; s__dispar |  |
| 1191 | 0 | 0 | 0 | 0 | 0 | 0 | 0 | 1 | 0 | k__Bacteria; p__Proteobacteria; c__Betaproteobacteria; o__Burkholderiales; f__Comamonadaceae |  |
| 161 | 0 | 0 | 0 | 0 | 0 | 0 | 0 | 2 | 0 | k__Bacteria; p__Firmicutes; c__Bacilli; o__Lactobacillales; f__Streptococcaceae; g__Streptococcus; s__ |  |
| 511 | 0 | 0 | 0 | 0 | 0 | 0 | 0 | 1 | 0 | k__Bacteria; p__Fusobacteria; c__Fusobacteriia; o__Fusobacteriales; f__Leptotrichiaceae; g__Leptotrichia; s__ |  |
| 190 | 0 | 0 | 0 | 0 | 0 | 0 | 0 | 1 | 0 | k__Bacteria; p__Bacteroidetes; c__Bacteroidia; o__Bacteroidales; f__Prevotellaceae; g__Prevotella; s__ |  |
| 168 | 0 | 0 | 0 | 0 | 0 | 0 | 1 | 0 | 0 | k__Bacteria; p__Actinobacteria; c__Actinobacteria; o__Actinomycetales; f__Micrococcaceae; g__Rothia; s__mucilaginosa |  |
| 178 | 0 | 0 | 0 | 0 | 0 | 0 | 0 | 1 | 0 | k__Bacteria; p__Proteobacteria; c__Betaproteobacteria; o__Neisseriales; f__Neisseriaceae; g__Neisseria; s__ |  |
| 112 | 0 | 0 | 0 | 0 | 0 | 0 | 0 | 1 | 0 | k__Bacteria; p__Bacteroidetes; c__Bacteroidia; o__Bacteroidales; f__Porphyromonadaceae; g__Porphyromonas; s__endodontalis |  |
| 51 | 0 | 0 | 0 | 0 | 0 | 0 | 0 | 1 | 0 | k__Bacteria; p__Firmicutes; c__Bacilli; o__Lactobacillales; f__Streptococcaceae; g__Streptococcus; s__ |  |
| 67 | 0 | 0 | 0 | 0 | 0 | 0 | 1 | 0 | 0 | k__Bacteria; p__Firmicutes; c__Bacilli; o__Bacillales; f__Staphylococcaceae; g__Staphylococcus; s__epidermidis |  |
| 392 | 0 | 0 | 0 | 0 | 0 | 0 | 0 | 1 | 0 | k__Bacteria; p__TM7; c__TM7-3; o__CW040; f__; g__; s__ |  |
| 142 | 0 | 0 | 0 | 0 | 0 | 0 | 0 | 1 | 0 | k__Bacteria; p__Proteobacteria; c__Betaproteobacteria; o__Burkholderiales; f__Burkholderiaceae; g__Lautropia; s__ |  |
| 129 | 0 | 0 | 0 | 0 | 0 | 0 | 1 | 0 | 0 | k__Bacteria; p__Firmicutes; c__Bacilli; o__Lactobacillales; f__Streptococcaceae; g__Streptococcus; s__ |  |
| 78 | 0 | 0 | 0 | 0 | 0 | 0 | 0 | 1 | 0 | k__Bacteria; p__Proteobacteria; c__Betaproteobacteria; o__Neisseriales; f__Neisseriaceae; g__Neisseria; s__cinerea |  |
| 266 | 0 | 0 | 0 | 0 | 0 | 0 | 0 | 2 | 0 | k__Bacteria; p__Firmicutes; c__Clostridia; o__Clostridiales; f__Veillonellaceae; g__Selenomonas; s__noxia |  |
| 665 | 0 | 0 | 0 | 0 | 0 | 0 | 0 | 1 | 0 | k__Bacteria; p__Bacteroidetes; c__Flavobacteriia; o__Flavobacteriales; f__Flavobacteriaceae; g__Capnocytophaga; s__ |  |
| 2786 | 0 | 0 | 0 | 0 | 0 | 0 | 4 | 0 | 0 | k__Bacteria; p__Firmicutes; c__Clostridia; o__Clostridiales; f__Clostridiaceae; g__Caloramator; s__ |  |
| 1238 | 0 | 0 | 0 | 0 | 0 | 0 | 13 | 0 | 0 | k__Bacteria; p__Firmicutes; c__Clostridia; o__Clostridiales; f__; g__; s__ |  |
| 1704 | 0 | 0 | 0 | 0 | 0 | 0 | 0 | 1 | 0 | k__Bacteria; p__Bacteroidetes; c__Bacteroidia; o__Bacteroidales; f__Porphyromonadaceae; g__Porphyromonas; s__ |  |
| 277 | 0 | 0 | 0 | 0 | 0 | 0 | 0 | 2 | 0 | k__Bacteria; p__Fusobacteria; c__Fusobacteriia; o__Fusobacteriales; f__Leptotrichiaceae; g__Leptotrichia; s__ |  |
| 102 | 0 | 0 | 0 | 0 | 0 | 0 | 8 | 0 | 0 | k__Bacteria; p__Cyanobacteria; c__Chloroplast; o__Streptophyta; f__; g__; s__ |  |
| 34 | 0 | 0 | 0 | 0 | 0 | 0 | 1 | 0 | 0 | k__Bacteria; p__Proteobacteria; c__Gammaproteobacteria; o__Pasteurellales; f__Pasteurellaceae; g__Haemophilus; s__parainfluenzae |  |

Abbreviations: ASVs, Amplicon Sequence Variants; NSC, normal saline control; NTC, no*-*template control;
