# Supplementary material for: Distinct Microbiomes of Gut and Saliva in Patients With Systemic Lupus Erythematous and Clinical Associations
Source: Front Immunol. 2021 Jul 1;12:626217. doi: 10.3389/fimmu.2021.626217 (PMC8281017; doi:10.3389/fimmu.2021.626217)
Supplement: Supplementary Figure 1 — Bacterial richness and diversity in feces samples of subgroups. (A) Bacterial richness and diversity index compared in fecal samples among LDAF, HDAF and HCF; (B) Bacterial richness and diversity index compared in fecal samples among MildF, ModerateF and SevereF. Statistically significant comparisons after the Wilcoxon rank-sum test and Benjamini–Hochberg false discovery rate (FDR) correction between groups are denoted as *0.05; ** < 0.01; and *** < 0.001. HCF, HC feces; HDAF, High Disease activity feces; LDAF, Low disease activity feces; MildF, mild feces; ModerateF, moderate feces; SevereF, severe feces. [file DataSheet_1.zip › Table S5 Presence of bacterial genus in SLEF and SLES.doc]

**Table S5. Presence of bacterial genus in SLEF and SLES**

| **Number** | **Only in SLEF** | **Only in SLES** | **Shared by SLEF and SLES** |
| --- | --- | --- | --- |
| 1 | [Eubacterium] | Abiotrophia | Acholeplasma |
| 2 | Actinobaculum | Agrobacterium | Achromobacter |
| 3 | Adlercreutzia | Alloiococcus | Acinetobacter |
| 4 | Aeromicrobium | Christensenella | Actinobacillus |
| 5 | Akkermansia | Dehalobacterium | Actinomyces |
| 6 | Butyricimonas | Desulfobulbus | Aerococcus |
| 7 | Catenibacterium | Desulfomicrobium | Aggregatibacter |
| 8 | Eggerthella | Flexispira | Alcaligenes |
| 9 | Erwinia | Geobacillus | Anaerococcus |
| 10 | Flavobacterium | Kingella | Anaerostipes |
| 11 | Flectobacillus | Mogibacterium | Anaerotruncus |
| 12 | Gallicola | Moraxella | Anaerovorax |
| 13 | Halothiobacillus | Myroides | Anoxybacillus |
| 14 | Helcococcus | Nitrospira | Arcanobacterium |
| 15 | Holdemania | Paludibacter | Atopobium |
| 16 | Jonquetella | Propionivibrio | Bacillus |
| 17 | Kaistobacter | Pseudoramibacter_Eubacterium | Bacteroides |
| 18 | Lachnobacterium | Rhodoplanes | Bifidobacterium |
| 19 | Magnetospirillum | Rubrobacter | Bilophila |
| 20 | Oscillospira | Schwartzia | Blautia |
| 21 | Paracoccus | Sharpea | Brachybacterium |
| 22 | Paraprevotella | Tannerella | Bulleidia |
| 23 | Pediococcus |  | Butyrivibrio |
| 24 | Plesiomonas |  | Caldicoprobacter |
| 25 | Renibacterium |  | Campylobacter |
| 26 | Sphingomonas |  | Capnocytophaga |
| 27 | Sporanaerobacter |  | Cardiobacterium |
| 28 | Ureaplasma |  | Catonella |
| 29 |  |  | Chryseobacterium |
| 30 |  |  | Clostridium |
| 31 |  |  | Collinsella |
| 32 |  |  | Comamonas |
| 33 |  |  | Coprococcus |
| 34 |  |  | Corynebacterium |
| 35 |  |  | Cupriavidus |
| 36 |  |  | Deinococcus |
| 37 |  |  | Delftia |
| 38 |  |  | Desulfovibrio |
| 39 |  |  | Dialister |
| 40 |  |  | Dorea |
| 41 |  |  | Eikenella |
| 42 |  |  | Enhydrobacter |
| 43 |  |  | Enterococcus |
| 44 |  |  | Facklamia |
| 45 |  |  | Faecalibacterium |
| 46 |  |  | Filifactor |
| 47 |  |  | Finegoldia |
| 48 |  |  | Fusobacterium |
| 49 |  |  | Gemella |
| 50 |  |  | Gordonia |
| 51 |  |  | Granulicatella |
| 52 |  |  | Haemophilus |
| 53 |  |  | Halomonas |
| 54 |  |  | Hydrogenophilus |
| 55 |  |  | Klebsiella |
| 56 |  |  | Lachnospira |
| 57 |  |  | Lactobacillus |
| 58 |  |  | Lactococcus |
| 59 |  |  | Lautropia |
| 60 |  |  | Leptotrichia |
| 61 |  |  | Lysinibacillus |
| 62 |  |  | Megamonas |
| 63 |  |  | Megasphaera |
| 64 |  |  | Meiothermus |
| 65 |  |  | Micrococcus |
| 66 |  |  | Mobiluncus |
| 67 |  |  | Morganella |
| 68 |  |  | Moryella |
| 69 |  |  | Mycoplasma |
| 70 |  |  | Neisseria |
| 71 |  |  | Ochrobactrum |
| 72 |  |  | Odoribacter |
| 73 |  |  | Oribacterium |
| 74 |  |  | Parabacteroides |
| 75 |  |  | Parvimonas |
| 76 |  |  | Peptococcus |
| 77 |  |  | Peptoniphilus |
| 78 |  |  | Peptostreptococcus |
| 79 |  |  | Phascolarctobacterium |
| 80 |  |  | Porphyromonas |
| 81 |  |  | Prevotella |
| 82 |  |  | Propionibacterium |
| 83 |  |  | Proteus |
| 84 |  |  | Providencia |
| 85 |  |  | Pseudomonas |
| 86 |  |  | Pyramidobacter |
| 87 |  |  | Ralstonia |
| 88 |  |  | Roseburia |
| 89 |  |  | Rothia |
| 90 |  |  | Ruminococcus |
| 91 |  |  | Scardovia |
| 92 |  |  | Selenomonas |
| 93 |  |  | Serratia |
| 94 |  |  | Slackia |
| 95 |  |  | Sneathia |
| 96 |  |  | Sphaerochaeta |
| 97 |  |  | Staphylococcus |
| 98 |  |  | Streptococcus |
| 99 |  |  | Sutterella |
| 100 |  |  | Tepidimicrobium |
| 101 |  |  | Thermus |
| 102 |  |  | Treponema |
| 103 |  |  | Veillonella |
| 104 |  |  | Virgibacillus |

Abbreviation: systemic lupus erythematous feces, SLEF; systemic lupus erythematous salivary, SLES
