# Supplementary material for: Distinct Microbiomes of Gut and Saliva in Patients With Systemic Lupus Erythematous and Clinical Associations
Source: Front Immunol. 2021 Jul 1;12:626217. doi: 10.3389/fimmu.2021.626217 (PMC8281017; doi:10.3389/fimmu.2021.626217)
Supplement: Supplementary Figure 1 — Bacterial richness and diversity in feces samples of subgroups. (A) Bacterial richness and diversity index compared in fecal samples among LDAF, HDAF and HCF; (B) Bacterial richness and diversity index compared in fecal samples among MildF, ModerateF and SevereF. Statistically significant comparisons after the Wilcoxon rank-sum test and Benjamini–Hochberg false discovery rate (FDR) correction between groups are denoted as *0.05; ** < 0.01; and *** < 0.001. HCF, HC feces; HDAF, High Disease activity feces; LDAF, Low disease activity feces; MildF, mild feces; ModerateF, moderate feces; SevereF, severe feces. [file DataSheet_1.zip › Table S6 Presence of bacterial genus in HCF and HCS.doc]

**Table S6. Presence of bacterial genus in HCF and HCS**

| **Number** | **HCF only** | **HCS only** | **Shared by HCF and HCS** |
| --- | --- | --- | --- |
| 1 | [Eubacterium] | Acetobacter | Abiotrophia |
| 2 | Akkermansia | Acholeplasma | Acinetobacter |
| 3 | Alcaligenes | Achromobacter | Actinobacillus |
| 4 | Amaricoccus | Aeromicrobium | Actinomyces |
| 5 | Anaerostipes | Alloiococcus | Adlercreutzia |
| 6 | Anaerotruncus | Anaerovorax | Aggregatibacter |
| 7 | Butyricimonas | Arcanobacterium | Agrobacterium |
| 8 | Catenibacterium | Blastomonas | Anaerococcus |
| 9 | Christensenella | Brachybacterium | Atopobium |
| 10 | Coprobacillus | Brevundimonas | Bacillus |
| 11 | Dehalobacterium | Butyrivibrio | Bacteroides |
| 12 | Dorea | Capnocytophaga | Bifidobacterium |
| 13 | Eggerthella | Catonella | Bilophila |
| 14 | Finegoldia | Chryseobacterium | Blautia |
| 15 | Holdemania | Cloacibacterium | Bulleidia |
| 16 | Lachnobacterium | Dechloromonas | Campylobacter |
| 17 | Morganella | Deinococcus | Cardiobacterium |
| 18 | Oligella | Delftia | Clostridium |
| 19 | Oxalobacter | Desulfobulbus | Collinsella |
| 20 | Parabacteroides | Desulfomicrobium | Comamonas |
| 21 | Pediococcus | Devosia | Coprococcus |
| 22 | Peptoniphilus | Enhydrobacter | Corynebacterium |
| 23 | Plesiomonas | Erwinia | Desulfovibrio |
| 24 | Proteus | Fervidobacterium | Dialister |
| 25 | Sneathia | Filifactor | Eikenella |
| 26 | Succinivibrio | Flexispira | Enterococcus |
| 27 | Turicibacter | Gordonia | Faecalibacterium |
| 28 |  | Helicobacter | Fusobacterium |
| 29 |  | Kingella | Gemella |
| 30 |  | Limnohabitans | Granulicatella |
| 31 |  | Marinococcus | Haemophilus |
| 32 |  | Methylibium | Hydrogenophilus |
| 33 |  | Methylobacterium | Klebsiella |
| 34 |  | Methyloversatilis | Lachnospira |
| 35 |  | Microbispora | Lactobacillus |
| 36 |  | Micrococcus | Lactococcus |
| 37 |  | Moraxella | Lautropia |
| 38 |  | Mycoplana | Leptotrichia |
| 39 |  | Mycoplasma | Lysinibacillus |
| 40 |  | Myroides | Megamonas |
| 41 |  | Nesterenkonia | Megasphaera |
| 42 |  | Nevskia | Meiothermus |
| 43 |  | Novosphingobium | Mobiluncus |
| 44 |  | Ochrobactrum | Mogibacterium |
| 45 |  | Paludibacter | Moryella |
| 46 |  | Paucibacter | Neisseria |
| 47 |  | Peptococcus | Odoribacter |
| 48 |  | Phenylobacterium | Oribacterium |
| 49 |  | Planomicrobium | Oscillospira |
| 50 |  | Propionivibrio | Paracoccus |
| 51 |  | Renibacterium | Paraprevotella |
| 52 |  | Rhodoplanes | Parvimonas |
| 53 |  | Rubrobacter | Peptostreptococcus |
| 54 |  | Sphaerochaeta | Phascolarctobacterium |
| 55 |  | Tannerella | Porphyromonas |
| 56 |  | Ureaplasma | Prevotella |
| 57 |  | Virgibacillus | Propionibacterium |
| 58 |  |  | Providencia |
| 59 |  |  | Pseudomonas |
| 60 |  |  | Pseudoramibacter_Eubacterium |
| 61 |  |  | Pyramidobacter |
| 62 |  |  | Ralstonia |
| 63 |  |  | Ramlibacter |
| 64 |  |  | Roseburia |
| 65 |  |  | Rothia |
| 66 |  |  | Ruminococcus |
| 67 |  |  | Scardovia |
| 68 |  |  | Schwartzia |
| 69 |  |  | Selenomonas |
| 70 |  |  | Serratia |
| 71 |  |  | Sharpea |
| 72 |  |  | Slackia |
| 73 |  |  | Sphingobium |
| 74 |  |  | Sphingomonas |
| 75 |  |  | Staphylococcus |
| 76 |  |  | Stenotrophomonas |
| 77 |  |  | Streptococcus |
| 78 |  |  | Sutterella |
| 79 |  |  | Tepidimicrobium |
| 80 |  |  | Thermus |
| 81 |  |  | Treponema |
| 82 |  |  | Variovorax |
| 83 |  |  | Veillonella |

Abbreviation: HCF, HC feces; HCS, HC saliva
